# Halting ErbB-2 isoforms retrograde transport to the nucleus as a new theragnostic approach for triple-negative breast cancer

**DOI:** 10.1038/s41419-022-04855-0

**Published:** 2022-05-09

**Authors:** Santiago Madera, Franco Izzo, María F. Chervo, Agustina Dupont, Violeta A. Chiauzzi, Sofia Bruni, Ezequiel Petrillo, Sharon S. Merin, Mara De Martino, Diego Montero, Claudio Levit, Gabriel Lebersztein, Fabiana Anfuso, Agustina Roldán Deamicis, María F. Mercogliano, Cecilia J. Proietti, Roxana Schillaci, Patricia V. Elizalde, Rosalía I. Cordo Russo

**Affiliations:** 1 Laboratory of Molecular Mechanisms of Carcinogenesis and Molecular Endocrinology, Instituto de Biología y Medicina Experimental (IBYME), CONICET, Vuelta de Obligado 2490, C1428ADN Buenos Aires, Argentina; 2 New York Genome Center, New York, NY USA; 3 Meyer Cancer Center, Weill Cornell Medicine, New York, NY USA; 4 Universidad de Buenos Aires (UBA), Facultad de Ciencias Exactas y Naturales, Departamento de Fisiología, Biología Molecular y Celular and CONICET-UBA, Instituto de Fisiología, Biología Molecular y Neurociencias (IFIBYNE), C1428EHA Buenos Aires, Argentina; 5 Department of Radiation Oncology, Weill Cornell Medicine, New York, NY USA; 6 Servicio de Ginecología, Sanatorio Sagrado Corazón, Buenos Aires, Argentina

**Keywords:** Breast cancer, Protein translocation, Oncogenes, Nuclear transport, Targeted therapies

## Abstract

Triple-negative breast cancer (TNBC) is clinically defined by the absence of estrogen and progesterone receptors and the lack of membrane overexpression or gene amplification of receptor tyrosine kinase ErbB-2/HER2. Due to TNBC heterogeneity, clinical biomarkers and targeted therapies for this disease remain elusive. We demonstrated that ErbB-2 is localized in the nucleus (NErbB-2) of TNBC cells and primary tumors, from where it drives growth. We also discovered that TNBC expresses both wild-type ErbB-2 (WTErbB-2) and alternative ErbB-2 isoform c (ErbB-2c). Here, we revealed that the inhibitors of the retrograde transport Retro-2 and its cyclic derivative Retro-2.1 evict both WTErbB-2 and ErbB-2c from the nucleus of BC cells and tumors. Using BC cells from several molecular subtypes, as well as normal breast cells, we demonstrated that Retro-2 specifically blocks proliferation of BC cells expressing NErbB-2. Importantly, Retro-2 eviction of both ErbB-2 isoforms from the nucleus resulted in a striking growth abrogation in multiple TNBC preclinical models, including tumor explants and xenografts. Our mechanistic studies in TNBC cells revealed that Retro-2 induces a differential accumulation of WTErbB-2 at the early endosomes and the plasma membrane, and of ErbB-2c at the Golgi, shedding new light both on Retro-2 action on endogenous protein cargoes undergoing retrograde transport, and on the biology of ErbB-2 splicing variants. In addition, we revealed that the presence of a functional signal peptide and a nuclear export signal (NES), both located at the N-terminus of WTErbB-2, and absent in ErbB-2c, accounts for the differential subcellular distribution of ErbB-2 isoforms upon Retro-2 treatment. Our present discoveries provide evidence for the rational repurposing of Retro-2 as a novel therapeutic agent for TNBC.

## Introduction

Triple-negative breast cancer (TNBC) is clinically defined by the absence of estrogen and progesterone receptors and lack of overexpression at the plasma membrane (PM) of ErbB-2/HER2 (MErbB-2), a member of the ErbB family of tyrosine kinase receptors, or of ERBB2 gene amplification. MErbB-2 overexpression/gene amplification accounts for the ErbB-2-positive BC subtype, whose growth is driven by MErbB-2 activation of downstream pathways [1]. We and others demonstrated that MErbB-2 also migrates to the nucleus (NErbB-2) in ErbB-2-positive BC. NErbB-2 is recruited to promoters/enhancers of target genes where it functions as a transcriptional regulator to induce resistance to anti-MErbB-2 therapies and promote growth and metastasis [2–8]. Unexpectedly, we found that TNBC expresses NErbB-2 and identified it as a biomarker of poor clinical outcome [9]. Furthermore, we revealed that nuclear wild-type ErbB-2 isoform (WTErbB-2), encoded by the canonical transcript T1, and our recently identified nuclear ErbB-2 isoform c (ErbB-2c), encoded by transcript T3, induce TNBC growth via their function as transcriptional regulators [9]. TNBC is a heterogeneous disease displaying several subtypes: basal-like 1 (BL1), basal-like 2 (BL2), mesenchymal (M), and luminal androgen receptor (LAR) [10]. Clinical biomarkers and targeted therapies for TNBC remain elusive, so chemotherapy has been the standard of care. Recently, two immune-checkpoint inhibitors, which take the brakes off the antitumor immune responses, were approved for specific and poor outcome TNBCs [11], highlighting the necessity to develop precision medicine tools for this disease.

ErbB-2 traffics to the nucleus via retrograde transport [12]. It is internalized from the PM by endocytosis and then sorted to early endosomes (EE) [13]. ErbB-2 translocation from the EE to the trans-Golgi network (TGN) occurs by microtubule-dependent movement by interacting with dynein and membrane fusion with the Golgi via syntaxin-6 (ref. [14]). ErbB-2 transport to the nucleus may occur by its release from the endoplasmic reticulum (ER) into the cytosol (ERAD pathway) or by release of membrane-bound ErbB-2 from the ER into the nucleoplasm (INTERNET pathway) [15, 16]. Regardless of the pathway, the Sec61 translocon mediates ErbB-2 export from the ER. NErbB-2 import occurs through interaction of its nuclear localization sequence (NLS) with importin β1 (ref. [13]).

Exogenous toxins and viruses hijack the retrograde pathway to access the ER and exert their deleterious effects. The small molecule Retro-2 (R2) acts as a selective inhibitor of the retrotransport to the ER of plant toxin ricin and bacterial Shiga-like toxins (SLT) by trapping them in the EE [17]. R2 spontaneously cyclizes to its dihydroquinazolinone derivative Retro-2^cycl^, which is the active compound [18]. Consequently, R2 cyclic compounds with improved activity were developed [19]. R2 and its cyclic derivatives also block infection by several viruses, including SARs-CoV-2 (ref. [19, 20]). R2 affects the subcellular localization of syntaxin-5, a Golgi protein involved in EE-to-TGN retrotransport [17, 21]. Recent studies revealed that R2 actually affects ER function by targeting the ER exit site component Sec16A, which retains syntaxin-5 at the ER and disrupts trafficking events at the EE/TGN interface [22]. In the frame of our findings that both nuclear WTErbB-2 and nuclear ErbB-2c induce TNBC growth with differential oncogenic potential and the discovery that ErbB-2 traffics to the nucleus by retrograde transport, we explored whether inhibition of the retrograde route with R2 modulates subcellular localization of ErbB-2 isoforms. We here reveal that R2 evicts ErbB-2 from the nucleus, accumulates WTErbB-2 and ErbB-2c in different intracellular compartments, and abrogates in vitro and in vivo TNBC proliferation. Our results highlight repurposing R2 as a targeted therapy for TNBC.

## Results

### R2 evicts WTErbB-2 and ErbB-2c from the nucleus of BC cells

**Fig. 1:**
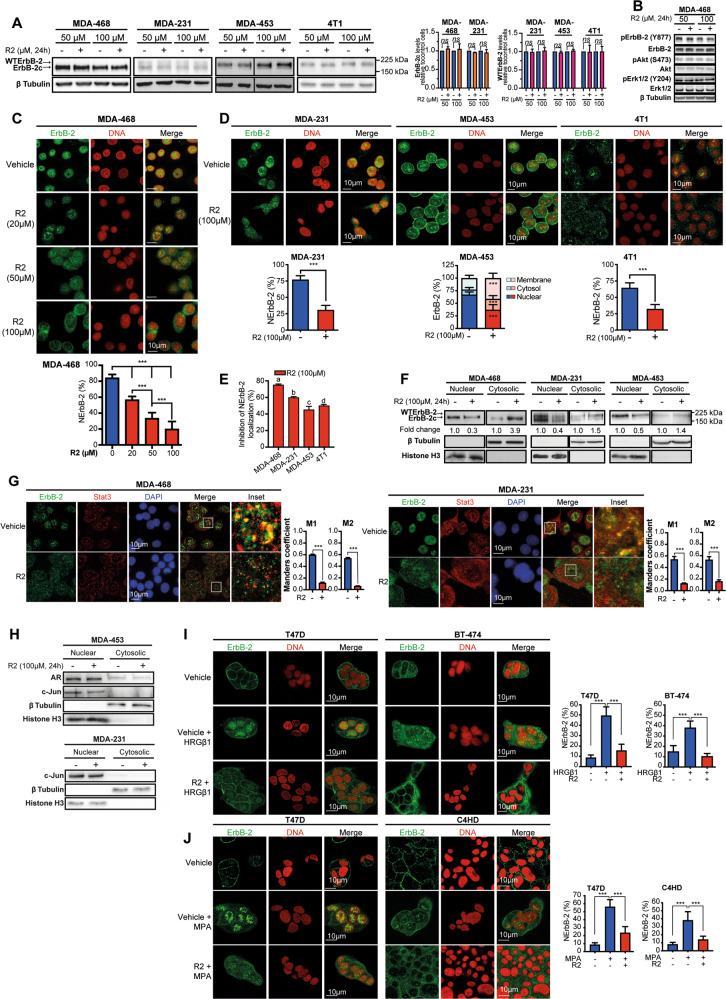
R2 evicts WTErbB-2 and ErbB-2c from the nucleus of BC cells. **A** Left: representative WB of ErbB-2 expression. Right: signal intensities of ErbB-2c and WTErbB-2 were analyzed by densitometry from three independent WBs performed as indicated. Fold change was calculated by normalizing the absolute levels of each ErbB-2 isoform to those of β tubulin, setting the value of vehicle-treated cells to 1. **B** Representative WB of cell lysates with the indicated phosphospecific or total antibodies. **C**, **D** ErbB-2 immunofluorescence (IF) in cells treated with R2 or vehicle (24 h). Bottom panels: quantitative analysis of ErbB-2 subcellular localization. Fluorescence intensity of nuclear, cytosolic, and membrane ErbB-2 was quantified and is plotted as percentage (mean ± SD, *n* = 50 per group) relative to total ErbB-2 in each cell. **E** Inhibition of NErbB-2 localization in cells from **C** to **D** (mean ± SEM). For c and d vs a: *P* < 0.001; for c and a vs b: *P* < 0.01; for d vs b: *P* < 0.05. **F** Nuclear and cytosolic lysates were analyzed by WB. Fold change was calculated for each compartment by normalizing ErbB-2 levels in treated vs control cells (value set to 1). **G** ErbB-2 and Stat3 were localized by IF and confocal microscopy in cells treated as in **D**. Merged images show colocalization in yellow. The insets show boxed areas in detail. Right: quantitative analysis of colocalization with Manders’ coefficients (M1 and M2, mean ± SEM, *n* = 50 per group). **H** Subcellular distributions of AR and c-Jun evaluated as in **F**. **I**, **J** Cells were pretreated with R2 (100 µM) or vehicle for 24 h and then treated with HRGβ1 (60 min) (**I**), or MPA (90 min) (**J**). ErbB-2 localization and NErbB-2 levels are depicted as in **C**. *ns*: not significant, ***P < 0.001. For (**A**–**J**), *n* = 3. Original uncropped WB images are shown in Fig. S9.

Since ErbB-2 migrates to the nucleus through retrograde transport [12], we explored whether R2 evicts ErbB-2 from the nucleus of TNBC. We already reported that human TN cells MDA-453 (LAR subtype) express only WTErbB-2, MDA-468 (BL1 subtype) only ErbB-2c, and MDA-231 (M subtype) both WTErbB-2 and ErbB-2c (Fig. 1A; [9]). We here included murine 4T1 cells as a model of metastatic TNBC from the basal-like immune-suppressed subtype [23]. Expression of ErbB-2 noncanonical isoforms in murine cells remains poorly explored. Our in silico studies showed that only one coding murine ErbB-2 transcript was reported, corresponding to mouse WTErbB-2 ortholog (mWTErbB-2; Fig. S1A, S1B). mWTErbB-2 shows 87.5% total amino-acid identity with human WTErbB-2 (hWTErbB-2; Fig. S1C, S1D). Consistently, 4T1 cells only express mWTErbB-2 (Fig. 1A). Total hWTErbB-2, mWTErbB-2, or ErbB-2c levels were not affected by R2 in these cells (Fig. 1A). This is consistent with R2 inability to modulate the levels of SLT exogenous cargo in cancer cells [17]. We previously reported that ErbB-2 phosphorylation at tyrosine 877, a site at the kinase domain, is mandatory for NErbB-2 localization [9]. We found that neither constitutive tyrosine 877 phosphorylation nor activation of ErbB-2 downstream pathways, such as Akt and Erk1/2 MAPKs, were affected by R2 (Fig. 1B; Fig. S2). Immunofluorescence studies showed that up to 90% of ErbB-2 was constitutively located in the nucleus of cells expressing ErbB-2c (MDA-468) or both ErbB-2c and hWTErbB-2 (MDA-231) (Fig. 1C, D; [9]). In MDA-453 cells, displaying only hWTErbB-2, it is more abundant in the nucleus, but is also present, albeit at lower amounts, in the cytoplasm and PM (Fig. 1D). Most of mWTErbB-2 in 4T1 cells is in the nucleus, and the remaining amount in the cytoplasm with barely detectable levels at PM. R2 decreased NErbB-2 levels in a dose-dependent manner (Fig. 1C depicts the results in MDA-468). Similar effects in all TNBC lines confirmed R2 capacity to evict NErbB-2 (Fig. 1D). Interestingly, only in MDA-453 cells, R2 induced PM localization. TNBC cells with higher NErbB-2 levels (MDA-468, MDA-231) showed greater sensitivity to R2-induced NErbB-2 eviction (Fig. 1E). Subcellular fractionation and immunoblotting confirmed that R2 decreased nuclear levels of WTErbB-2 and ErbB-2c (Fig. 1F). These findings are consistent with R2 inhibition of SLT retrotransport to the TGN/Golgi. However, they differ from R2 lack of effect on well-studied endogenous retrograde cargoes [17].

ErbB-2 shares components of the retrotransport machinery that traffics from the ER to the nucleus with endogenous proteins that shuttle between the cytoplasm and the nucleus. Among said proteins are BC key players such as transcription factors (TFs), hormone receptors (HRs), which act as TFs in the nucleus and activate signaling in the cytoplasm, and proteins with dual functions, comparable to HRs, such as Stat3 (ref. [24–26]). Stat3, HRs, and TFs nuclear entrance is mediated by importin β1, which also associates with ErbB-2 during its ER-to-nucleus retrotransport. R2 effects on the passage of endogenous cargoes from the ER to the nucleus remain unexplored. We revealed that constitutive Stat3 nuclear presence was not affected by R2 (Fig. 1G). However, ErbB-2/Stat3 colocalization decreased due to R2 action on ErbB-2 (Fig. 1G). Androgen receptor (AR) expression defines the LAR-TNBC subtype and induces TNBC growth [27]. Under growth conditions, AR was mainly located in the nucleus of MDA-453 and R2 did not impact this scenario (Fig. 1H). We previously demonstrated that ErbB-2, Stat3, as well as c-Jun and c-Fos, members of the AP-1 factor, assemble a transcriptional complex at the cyclin D1 promoter in BC [6]. Here, we found that nuclear c-Jun presence also remained unaffected by R2 in TNBC cells (Fig. 1H). Since disruption of the ErbB-2/importin β1 complex abrogates NErbB-2 translocation [13], we hypothesized that high importin β1 abundance in TNBC favors NErbB-2 localization. Bioinformatics analysis using the Cancer Cell Line Encyclopedia (CCLE) showed higher importin β1 message levels in TNBC than in ErbB-2-positive cells (Fig. S3A). Interestingly, when refining our search to TNBC cells with NErbB-2 expression (NErbB-2+TN), we again found higher importin β1 levels in NErbB-2+TN cells (Fig. S3B).

Next, we validated R2 effects in other BC molecular subtypes. T47D cells, from the luminal subtype, express estrogen and progesterone receptors (PR) and low levels of MErbB-2 (ref. [28]). BT-474 and murine C4HD cells mimic the estrogen receptor- and PR-positive, MErbB-2-overexpressing subtype [28, 29]. We and others reported that in these lines, both heregulin β1 (HRGβ1), an ErbBs ligand, and progestins induced NErbB-2 translocation, which stimulated proliferation [30]. We now found that HRGβ1 and progestin effects were abrogated by R2 (Fig. 1I, J), supporting the role of retrotransport in NErbB-2 trafficking induced by steroid hormones and growth factors. Our findings also indicate that R2 inhibition of NErbB-2 localization is not related with modulation of ErbB-2 kinase activity or blockade of ErbB-2 canonical downstream signaling, but with R2 disruption of ErbB-2 retrotransport.

### Blockade of ErbB-2 retrotransport with R2 and its cyclic derivative Retro-2.1 inhibits TNBC growth

**Fig. 2:**
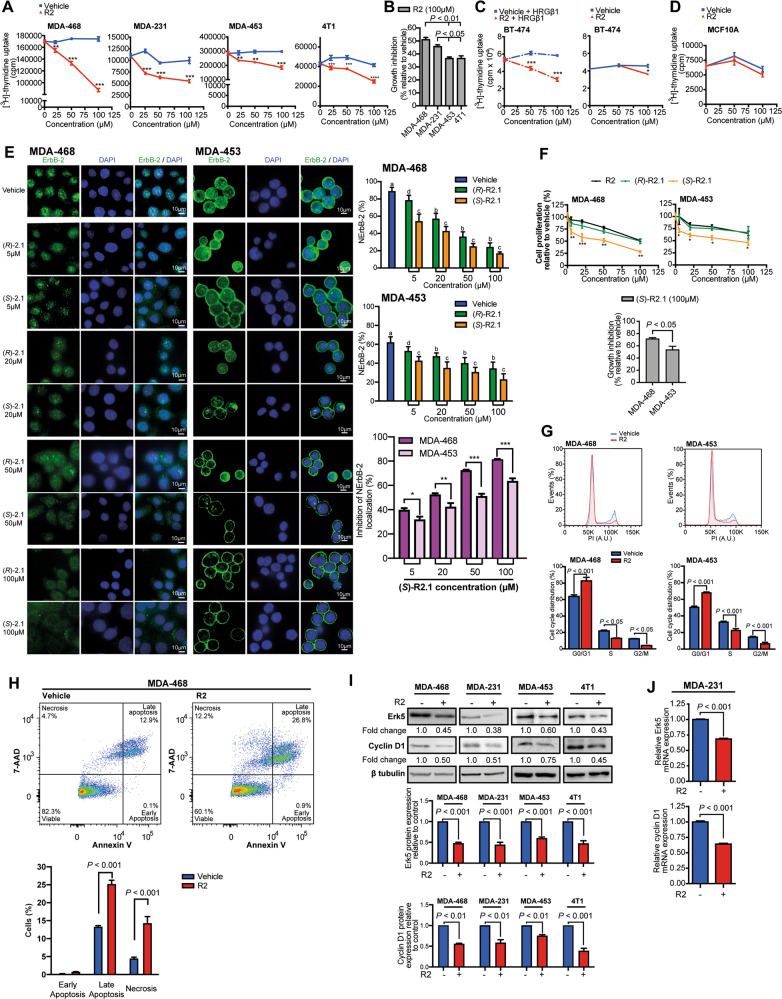
Blockade of ErbB-2 retrotransport with R2 and its cyclic derivative Retro-2.1 inhibits TNBC growth. **A** Proliferation was assayed by [^3^H]-thymidine uptake in cells treated as indicated for 24 h. A representative experiment is shown. **B** Growth inhibition of TNBC cells after R2 treatment. **C** BT-474 cells were pretreated with R2 or vehicle, and then treated or not with HRGβ1 (40 ng/ml, 24 h). Cell proliferation was assayed as in **A**. **D** MCF10A cell proliferation was assayed as in **A**. **E** R2 cyclic derivatives *(S)-*R2.1 and *(R)*-R2.1 evict ErbB-2 from the nucleus in TNBC cells. ErbB-2 IF in cells treated with *(S)-*R2.1, *(R)*-R2.1, or vehicle at the indicated concentrations for 24 h. Right: quantitative analysis of ErbB-2 subcellular localization. Fluorescence intensity of NErbB-2 was quantified and plotted as in Fig. 1C. For b and c vs a, and for c vs b and d, *P* < 0.001. Inhibition of NErbB-2 localization comparing *(S)-*R2.1 effects in MDA-468 and MDA-453 cells. **F** Proliferation was assayed in cells treated with R2, *(S)-*R2.1, or *(R)*-R2.1 at the indicated concentrations for 24 h. Data are shown as percentage of cell proliferation relative to vehicle-treated cells. Bottom: growth inhibition after *(S)-*R2.1 treatment. **G** Propidium iodide staining and flow cytometry in cells treated with R2 (100 µM) or vehicle for 48 h. **H** Annexin-V/7-aminoactinomycin D (7-AAD) labeling followed by flow cytometry was used to measure apoptosis in cells treated as in **G**. **I** WB analysis of Erk5 and cyclin D1 expression in cells treated as in **G**. Bottom: Fold- change values after densitometric normalization of bands to their corresponding β tubulin. **J** Erk5 and cyclin D1 mRNA levels were determined by RT-qPCR in cells treated with R2 (100 µM) or vehicle for 24 h. Relative mRNA levels were calculated by normalizing the absolute mRNA levels to GAPDH mRNA levels, and setting the value of control cells to 1. For (**A**–**J**), *n* = 3, mean ± SEM. *P < 0.05, **P < 0.01, ***P < 0.001.

Given NErbB-2 role as TNBC driver [9] and our discovery that retrotransport inhibition blocks NErbB-2 presence, we explored whether R2 affects TNBC proliferation. R2 abrogates growth of all TNBC lines (Fig. 2A) and it exerted higher effects in cells expressing higher NErbB-2 levels and ErbB-2c (MDA-468, MDA-231) (Fig. 2B). In the BT-474 model of HRGβ1-inducible NErbB-2 localization, R2 also abrogated proliferation stimulated by HRGβ1, highlighting the specificity of R2 action (Fig. 2C). No effects of R2 were found in unstimulated BT-474 cells, with low NErbB-2 expression, or in MCF10A breast cells lacking ErbB-2 (ref. [31]), further demonstrating R2 specificity to block proliferation of NErbB-2-positive cells (Fig. 2C, D). Retro-2.1 (R2.1), a R2 cyclic derivative, is synthesized as a racemate. Its *(S)*-enantiomer (*(S)-*R2.1) showed more potency as retrotransport inhibitor than the *(R)-*enantiomer (*(R)-*R2.1) (ref. [32]). We found that (*S*)-R2.1 induced higher NErbB-2 eviction and, consistently, stronger growth-inhibitory effects than (*R*)-R2.1 or R2 (Fig. 2E, F). ErbB-2c-expressing cells showed greater sensitivity to (*S*)-R2.1 effects (both at NErbB-2 localization and proliferation) than WTErbB-2-expressing cells (Fig. 2E, F). Our findings on the mechanisms of R2 inhibition of proliferation revealed that it induced cell-cycle arrest, as shown by an increase of the percentage of cells in G0/G1 phase and a reduction in S and G2/M (Fig. 2G). R2 also increased late apoptosis and necrosis (Fig. 2H). Furthermore, R2 decreased the levels of Erk5 (extracellular signal-regulated kinase 5) and cyclin D1 (Fig. 2I, J), genes transcriptionally regulated by NErbB-2 (ref. [3, 9]). Jointly, our findings show that R2 blockade of NErbB-2 retrotransport downregulates expression of proteins driving TNBC growth, arrests cell cycle, and induces cell death by apoptosis and necrosis.

### Inhibition of retrotransport with R2 abrogates growth of TNBC in ex vivo and in vivo preclinical models

**Fig. 3:**
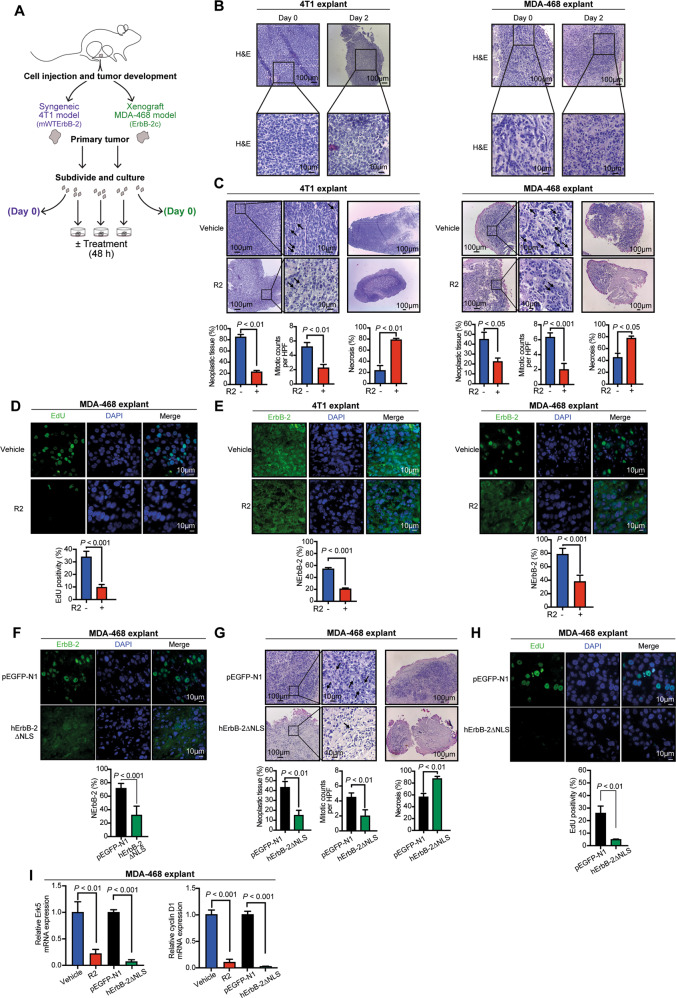
R2 inhibits growth of TNBC explants. **A** Schematic method for ex vivo culture of 4T1 or MDA-468 tumors (see “Materials and methods” for details). **B** Representative H&E staining of original, uncultured tissue (Day 0) and explants (Day 2). **C** H&E-stained histological sections of explants treated with R2 (100 µM) or vehicle. Tumor proliferation was quantified by mitotic counts (black arrows) and percentage of tumor necrosis was measured at lower magnification (mean ± SEM). **D** Representative images of EdU-labeled MDA-468 explants. Bottom: quantification of EdU staining (mean ± SD, *n* = 50 per group). **E** ErbB-2 IF in histological sections from explants. NErbB-2 levels are depicted as in Fig. 1C. **F** ErbB-2 IF in transfected explants as in **E. G**, **H** Tumor proliferation, necrosis, and EdU positivity from transfected explants depicted as in **C** and **D**. **I** Erk5 and cyclin D1 mRNA levels were determined and depicted as in Fig. 2J.

Compelling findings revealed NErbB-2 major role in BC, yet no drug able to evict ErbB-2 from the nucleus entered a clinical trial. R2 emerges as an attractive therapeutic agent for this purpose. Not only R2 lacks toxicity in vivo but it is also included in the pandemic response box, a library of compounds with antibacterial and antiviral properties [17, 20], favoring its repurposing against BC. As a preclinical model, we used tumor explants. As a model of exclusive WTErbB-2 expression, we selected murine 4T1 over MDA-453 cells because, although both lack ErbB-2c, 4T1 also lacks transcript T3. MDA-468 cells were our model displaying ErbB-2c. 4T1 and MDA-468 tumors developed in mice were excised and then cultured ex vivo as explants (Fig. 3A). Histopathological analyses demonstrated that tumor and stromal morphologies of explants were similar to those in the original, uncultured tissue (Fig. 3B). R2 treatment of 4T1 and MDA-468 explants resulted in decreased neoplastic tissue, lower mitotic figures, and higher necrosis than vehicle-treated ones (Fig. 3C). R2 growth inhibition was also evident in MDA-468 explants when assessing proliferation with EdU, a marker of DNA synthesis (Fig. 3D). R2 also decreased NErbB-2 levels in TNBC explants (Fig. 3E). Transfection of MDA-468 explants with our well-characterized human ErbB-2 NLS mutant (hErbB-2ΔNLS), which is unable to translocate to the nucleus and acts as dominant negative inhibitor of endogenous NErbB-2 migration [9], was used as another approach to block nuclear ErbB-2 localization. Consistently, NErbB-2 levels were reduced in hErbB-2ΔNLS-transfected explants (Fig. 3F; Fig. S4). hErbB-2ΔNLS-transfected explants showed similar growth inhibition to the one observed in R2-treated explants (Fig. 3G and H). In accordance with R2 effects in vitro, we also found that NErbB-2 eviction by R2 or hErbB-2∆NLS reduced Erk5 and CCND1 levels in MDA-468 explants (Fig. 3I).

**Fig. 4:**
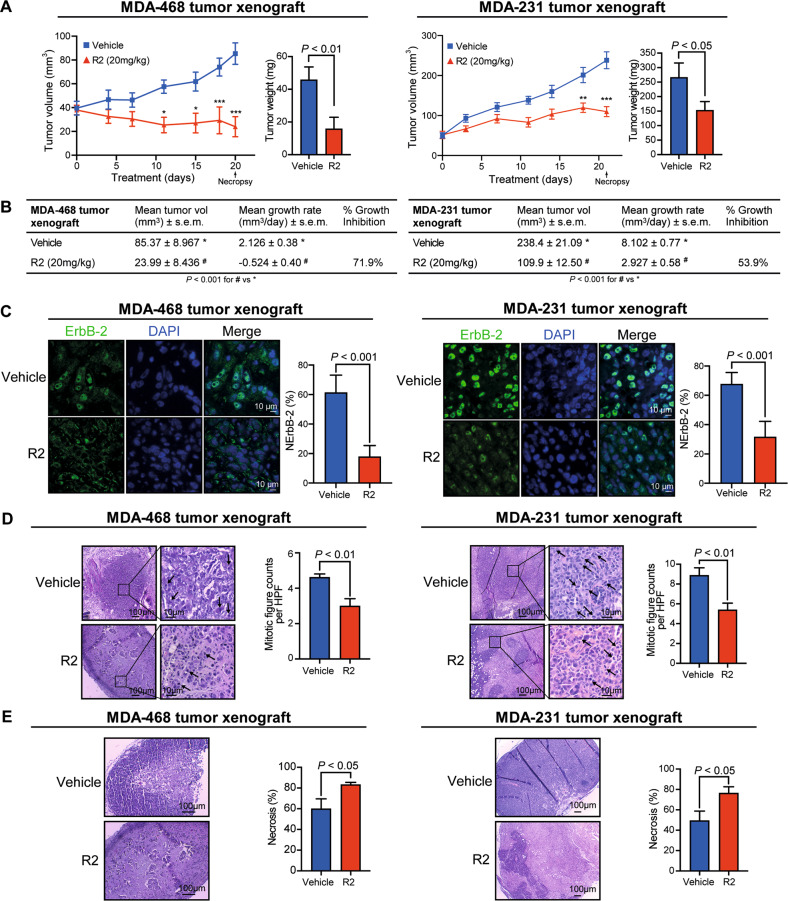
R2 abrogates TNBC tumor growth in vivo. **A** MDA-468 or MDA-231 tumor xenografts were injected as indicated in Materials and methods. Bar graphs depict tumor weight at necropsy. Mean±SEM, *n* = 6. *P < 0.05, **P < 0.01, ***P < 0.001. **B** Final tumor volumes, growth rates, and inhibition were calculated as stated in Materials and methods. **C** ErbB-2 IF in histological sections at necropsy. NErbB-2 levels depicted as in Fig. 1C. **D**, **E** H&E-stained histological sections. **D** Tumor proliferation was quantified by mitotic counts (black arrows). **E** Percentage of tumor necrosis was measured at lower magnification (mean ± SEM).

As another preclinical model, we used MDA-468 and MDA-231 tumor xenografts established in nude mice and injected intraperitoneally with R2 or vehicle. Volumes, growth rates, and weights of tumors from R2-injected mice were lower than those from vehicle-injected animals (Fig. 4A, B). NErbB-2 levels and mitotic figures per HPF were lower, and necrosis percentages higher in tumors from R2-treated animals than in tumors from control animals (Fig. 4C–E). As reported [17], R2 showed no toxicity in either mice organs or blood (Table S1; Fig. S5).

### R2 causes retention of WTErbB-2 and ErbB-2c in different intracellular compartments

**Fig. 5:**
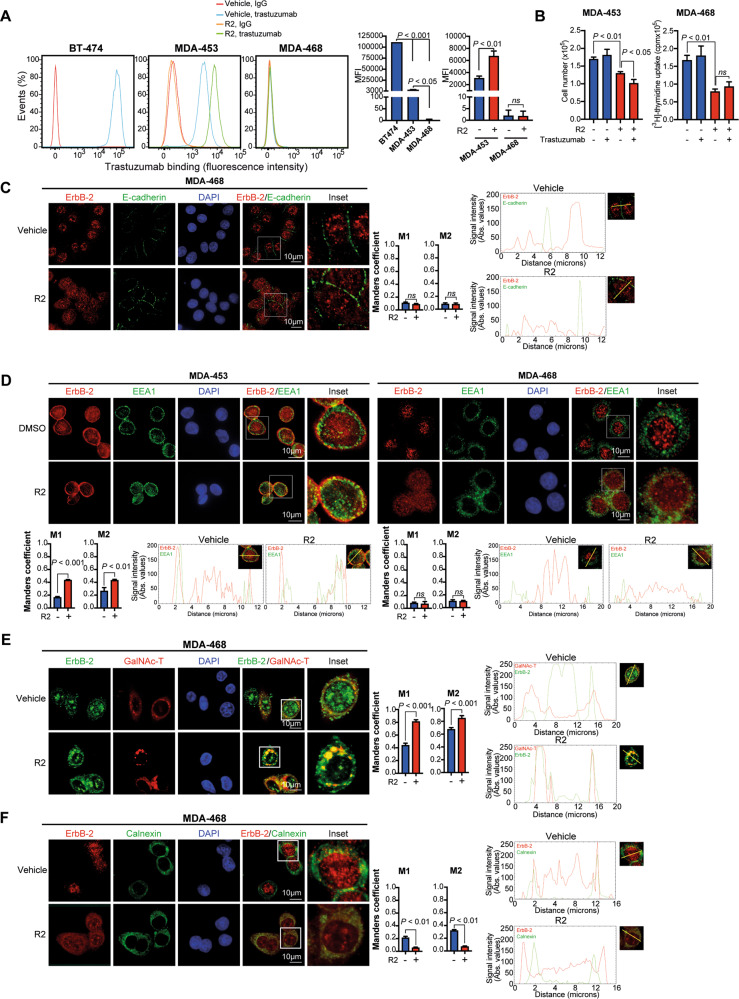
R2 causes retention of WTErbB-2 and ErbB-2c in different intracellular compartments. **A** Trastuzumab binding was determined in cells treated with R2 (100 µM) or vehicle for 24 h. Representative histograms. Quantification of trastuzumab binding by delta MFI (*n* = 2, mean ± SD). Left barplot: cells in control conditions (DMSO); right barplot: MFI of treated cells. **B** Cells were pretreated with R2 (100 µM) or vehicle and then with trastuzumab (10 µg/ml) for analysis of proliferation by cell count or [^3^H]-thymidine incorporation. **C**−**F**, Colocalization of ErbB-2 with E-cadherin (**C**), EEA1 (**D**), GalNAc-T-Cherry (**E**), and calnexin (**F**) was analyzed by IF in cells treated as in **A**. Colocalization analysis was performed as in Fig. 1G. The yellow line indicates the plane used for line profile generation (see Supplementary Materials and Methods). Experiments shown in (**B**–**F**): *n* = 3.

To identify in which intracellular compartment R2 altered ErbB-2 retrotransport, we first examined its effects on ErbB-2 expression at the PM. We studied MErbB-2 levels by binding of trastuzumab, an antibody that recognizes ErbB-2 extracellular domain IV [33]. While trastuzumab is used in the clinic for ErbB-2-positive BC, it has no antiproliferative effects on TNBC cells [34]. Consistent with reported MErbB-2 levels [9], flow cytometry showed strikingly lower trastuzumab binding in MDA-453 than in MErbB-2-positive BT-474 cells, and lack of trastuzumab binding in MDA-468 (Fig. 5A). R2 increased trastuzumab-binding capacity in MDA-453, indicating that it relocates WTErbB-2 to the PM. In contrast, R2 did not induce trastuzumab binding in MDA-468, displaying only ErbB-2c. Since ErbB-2c retains the trastuzumab-binding domain [9], our findings indicate that ErbB-2c never reaches the PM upon R2 treatment. The antiproliferative effects of R2 plus trastuzumab in MDA-453 were higher than those of R2 alone (Fig. 5B), reflecting WTErbB-2 presence at the PM. Therefore, this drug combination offers the possibility to block NErbB-2 and MErbB-2 growth effects. Contrastingly, R2 did not sensitize MDA-468 cells to trastuzumab effects (Fig. 5B). In addition, IF studies in MDA-468, both R2-treated and -untreated, showed no colocalization of ErbB-2c with the PM marker E-cadherin (Fig. 5C), confirming ErbB-2c absence at the PM either constitutively or after retrotransport inhibition. Since R2 arrests SLT trafficking in EE [17], we explored whether it modulates ErbB-2 colocalization with Early Endosome Antigen 1 (EEA1), an EE marker. Under growth conditions, we found EEA1/WTErbB-2 colocalization mostly in MDA-453 cell periphery, which was strongly enhanced by R2 (Fig. 5D). Fluorescence intensity profiling demonstrated higher overlap between WTErbB-2 and EEA1 signals in R2-treated compared with -untreated cells, further showing restraint of WTErbB-2 trafficking at the EE. We found neither constitutive nor R2-induced ErbB-2c/EEA1 colocalization in MDA-468 (Fig. 5D). This result, as opposed to that in MDA-453, suggests that EEs are not involved in ErbB-2c retrotranslocation. To gain insight into how R2 contributes to ErbB-2c delocalization from the nucleus, MDA-468 cells were transfected with a plasmid encoding N-acetylgalactosaminyltransferase (GalNAc-T, a Golgi marker) or immunostained with the ER marker calnexin. Low basal ErbB-2c levels were detected at the cytoplasm, which colocalized with Golgi or ER markers (Fig. 5E, F). R2 enhanced ErbB-2c/GalNAc-T colocalization, as determined by increased Manders correlation coefficients in R2-treated compared with -untreated cells (Fig. 5E). Contrastingly, R2 decreased ErbB-2c/calnexin colocalization (Fig. 5F). Altogether, these results indicate that R2 induces ErbB-2c accumulation in the Golgi, further preventing its sorting to the ER and to the nucleus of MDA-468 cells.

### ErbB-2 mRNA variant and isoform structures dictate their differential subcellular retention upon R2 treatment

**Fig. 6:**
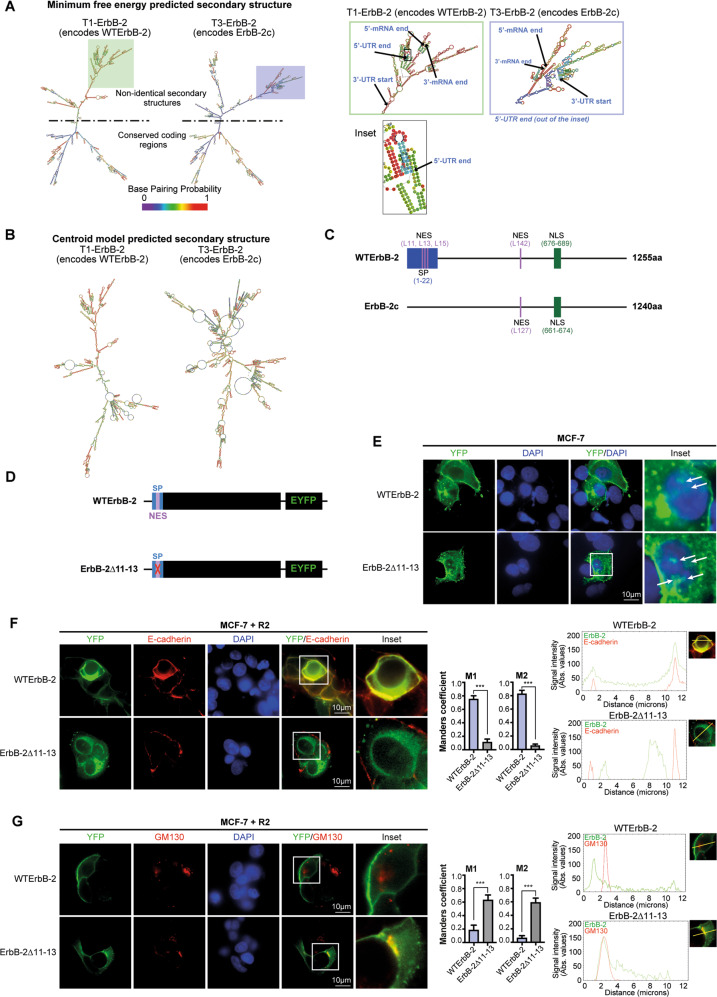
ErbB-2 mRNA variant and isoform structures dictate their differential subcellular retention upon R2 treatment. **A**, **B** In silico-predicted secondary structures of full-length T1 and T3 transcripts. (**A**) Minimum free energy and (**B**) centroid model predicted secondary structures. The right panel in **A** shows UTRs and the first region of CCDSs in detail. **C** Schematic representation of ErbB-2 isoforms. The position of the signal peptide (SP) in the N-terminus is shown, and the NLS and NES are also shown. **D** Schematic representation of WTErbB-2-pEYFP (WTErbB-2) vector and ErbB-2∆11–13-pEYFP (ErbB-2∆11–13) mutant. **E** ErbB-2 subcellular localization was visualized by direct fluorescence imaging (green) in MCF-7-transfected cells with WTErbB-2-pEYFP (WTErbB-2) or ErbB-2∆11–13-pEYFP (ErbB-2∆11–13) in growing-media conditions. White arrows indicate NErbB-2. **F**, **G** Colocalization of ErbB-2 vectors (WTErbB-2 or ErbB-2∆11–13) with E-cadherin (**F**) or GM130, a marker of Golgi complex (**G**), was analyzed by confocal microscopy in cells treated with R2 (100 µM, 24 h). Colocalization analysis was performed as in Fig. 5. The yellow line indicates the plane used for line profile generation (see Supplementary Materials and Methods). Experiments shown in (E–G): *n* = 3. ***P < 0.001.

Compelling evidence demonstrated that mRNAs 5′- and 3′-untranslated regions (UTRs), as well as consensus coding sequences (CCDSs), mediate post-transcriptional regulation of gene expression and determine protein levels and fate [35–38]. Therefore, we hypothesized that T1 and T3 structures define the fate of their encoded WTErbB-2 and ErbB-2c. We first performed in silico mRNA secondary structure analysis using ViennaRNA package [39]. The prediction from the minimum free energy and centroid models showed that the secondary structures from full-length T3 and T1 differ (Fig. 6A, B). Although part of T1 and T3 mRNA secondary structures are conserved in minimum free energy models, T3 showed a higher number of branches and loops in the region containing the UTRs and the first CCDSs’ nucleotides (Fig. 6A). The centroid model also showed more branches and loops for T3 (Fig. 6B). We then investigated the regions accounting for these dissimilarities. While T3 displays a different 5′UTR sequence than T1, both transcripts share the 3′UTR (Fig. S6A-B; [9]). The CCDSs of these transcripts were highly similar; however, the first 69 nucleotides of T1 differ from the initial 24 nucleotides of T3 (Fig. S6C). Consequently, T3 gives rise to ErbB-2c with the same C-termini as WTErbB-2 but a shorter N-termini, lacking the signal peptide (SP, [9]). Afterward, we performed an in silico protein sequence study. We focused on the balance between NLS and nuclear export signals (NES), which govern TFs’ nuclear localization [40]. The NLS previously identified in WTErbB-2 is conserved in ErbB-2c ([41], Fig. 6C); however, the NES responsible for its nuclear export remains unknown. Using NetNES 1.1 software [42], we found that both isoforms have a putative NES at Leu (L) 142 in WTErbB-2 and L127 in ErbB-2c. WTErbB-2 also presents a putative NES consisting of an L-rich motif (^11^LLLAL^15^, Fig. 6C, Fig. S7A). This differential NES is located within the hydrophobic domain of WTErbB-2 SP, required for translocation to the PM [43].

To investigate whether a functional SP and an extra NES play a role in the differential retention of ErbB-2 isoforms by R2, we generated a mutant of WTErbB-2 by deleting residues L11–L13 (ErbB-2Δ11–13), which resulted in the disruption of both the NES and the SP hydrophobic domain (Fig. 6D, Fig. S7B). Transfection of MCF-7 BC cells, which express low ErbB-2 levels, with WTErbB-2 vector showed that WTErbB-2 localized predominantly in the PM, but low levels were also found in the cytoplasm and nucleus (Fig. 6E). In ErbB-2Δ11–13-transfected cells, ErbB-2 expression at the PM was lower and localized predominantly in the cytoplasm and nucleus (Fig. 6E). These observations suggest that ErbB-2Δ11–13 mutant mimics ErbB-2c subcellular localization. Comparable activation of ErbB-2 and its downstream pathway Akt was observed in WTErbB-2- and ErbB-2Δ11–13-transfected cells, denoting that L11–L13 deletion did not affect ErbB-2 signaling (Fig. S8). Moreover, MCF-7 cells treated with R2 showed colocalization of E-cadherin with WTErbB-2, but not with ErbB-2Δ11–13, confirming ErbB-2Δ11–13 absence at the PM (Fig. 6F). R2 also led to ErbB-2Δ11–13 retention at the Golgi (Fig. 6G). These findings demonstrated that upon R2 treatment, ErbB-2∆11–13 fate resembled that of ErbB-2c, suggesting that the presence of a functional SP and a NES in WTErbB-2 accounts for the differential R2-induced subcellular retention of ErbB-2 isoforms.

## Discussion

**Fig. 7:**
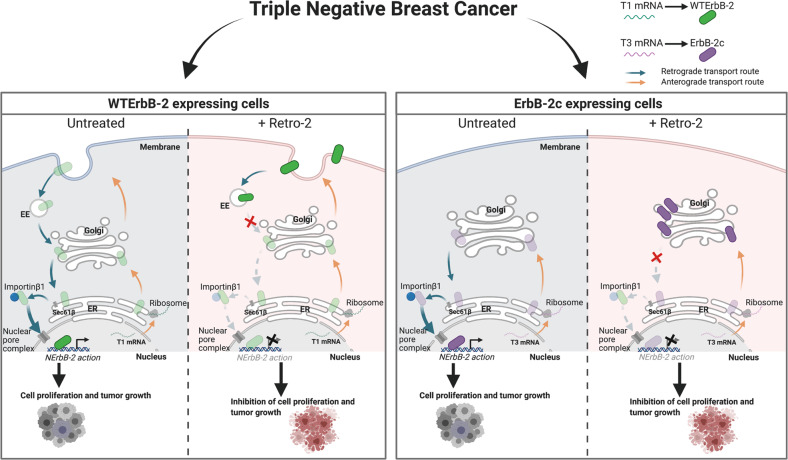
Model of R2 effects on ErbB-2 nuclear migration in TNBC. TNBC expresses at the nucleus either WTErbB-2, encoded by T1, or ErbB-2c, encoded by T3, or both. T1 and T3 are translated at ribosomes and WTErbB-2 and ErbB-2c enter the anterograde route where they undergo post-translational modifications to form their mature proteins. ErbB-2 isoforms traffic to the nucleus via the retrograde route, where they act as transcriptional regulators to induce TNBC growth. R2 blocks ErbB-2 retrotransport, leading to a differential accumulation of WTErbB-2 at EE and PM, and of ErbB-2c at the Golgi, and, consequently, inhibiting TNBC growth.

Our discovery that R2 blocks ErbB-2 retrotransport in TNBC, leading to a differential accumulation of WTErbB-2 at EE and the PM and of ErbB-2c at the Golgi, sheds new light on R2 action on endogenous protein cargoes (i.e., ErbB-2 isoforms) undergoing retrograde transport. These results also provide novel knowledge on the biology of ErbB-2 splicing variants, revealing that it is indeed the cargo itself which determines its final cellular location. Most importantly, R2 abrogates TNBC growth in vitro, ex vivo, and in vivo, supporting the rational of its repurposing as a novel therapeutic agent for TNBC. Figure 7 illustrates our proposed model of R2 action.

While the ability of R2 to block the retrograde trafficking of exogenous toxins and viruses is well acknowledged [19], its effects on endogenous cargoes remain almost unexplored and limited to specific sets of proteins that function as mediators of protein trafficking. R2 does not affect the trafficking of cation-independent mannose 6-phosphate receptor, which captures mannose 6-phosphate-tagged lysosomal enzymes and is then recycled to the TGN or of TGN protein 46 that mediates protein trafficking between the Golgi and the PM [17, 44]. The findings of R2 action on exogenous cargoes and our present discoveries on endogenous ErbB-2 isoforms support the exciting notion that R2 might selectively target cargoes whose localization, via retrotransport, drives either cell death, as toxins and viruses, or neoplastic transformation, such as ErbB-2 when ectopically located in the nucleus.

We are the first to study R2 effects on the passage of endogenous cargoes from the ER to the nucleus. We found that R2 blocks WTErbB-2 and ErbB-2c nuclear translocation, but does not prevent the nuclear entry of TFs, Stat3, AR, and c-Jun, mediated by importin β1, which is also a key player in ErbB-2 trafficking from the ER to the nucleus. At that point of our study, we felt tempted to interpret these results as a surprisingly selective action of R2 at such stage of the retrograde transport, allowing nuclear access only to proteins that mediate cell physiological functions, but we knew that further research was necessary to elucidate this puzzle. Indeed, our mechanistic studies of R2 action at cellular level provided a rational explanation. We found that R2 induces WTErbB-2 accumulation at the EEs and the PM, and ErbB-2c accumulation at the Golgi, highlighting that R2 retention of both ErbB-2 isoforms occurs at more distal cellular locations, therefore not compromising the ER-nucleus passage. Our findings also revealed that R2 may act as an inhibitor not only at the EE–Golgi interface, as reported [17], but also at the Golgi–ER interface.

What is clear from our work is that the final cellular fate of ErbB-2 upon R2 treatment is isoform-specific. In silico studies showed that T1 and T3 mRNA secondary structures vary in the region of the CCDSs coding for the SP and the differential NES we identified in WTErbB-2. Using site-directed mutagenesis, we revealed that a functional SP and a NES determine the subcellular-specific localization of ErbB-2 isoforms and thus the intracellular compartments at which they are retained by R2. T1 and T3 secondary structures also differ in their UTRs. Functional regions in 3′UTRs have been well studied but those in 5′UTRs remain less characterized. Secondary structure elements in 3′UTRs are recognized by RNA-binding proteins [45], which recruit effector proteins mediating local translation through the regulation of mRNA localization [46]. A deep study on T1 and T3 structure profiles is beyond the scope of our work. However, we hope our findings will encourage research on the functional regulatory sequences underlying splice events in ErbB-2 transcripts and the RNA-binding proteins involved in the process.

R2 showed an exquisite specificity to block the growth of BC expressing NErbB-2. In TNBC cells of different subtypes, R2 inhibition of in vitro proliferation correlates with its ability to evict ErbB-2 from the nucleus. R2 derivative (*S*)-R2.1 more effectively blocked both NErbB-2 localization and proliferation. A remarkable sensitivity to R2 and (*S*)-R2.1 was found in TNBC expressing ErbB-2c. This could be due to our previous discovery that ErbB-2c is a major oncogenic driver in TNBC [9]. Our studies in hormone receptor-positive BC models displaying low (T47D) or high (BT-474 and C4HD) levels of MErbB-2 revealed that when we induce NErbB-2 migration via stimulation with HRGβ1 or progestin, R2 is again able to block its nuclear migration. Furthermore, we proved that while R2 does not affect growth in ErbB-2-positive BC cells without NErbB-2 (BT-474), when we induced NErbB-2 migration, R2 displayed strong antiproliferative effects. R2 has no effect on proliferation in a model lacking ErbB-2 expression, normal breast MCF10A cells, further disclosing the specificity of its action. These findings are in agreement with reports showing that R2 does not block growth in normal or in cancer cells, where no ErbB-2 was found [20, 47–49]. One of our findings with major clinical impact is the discovery that enhanced membrane WTErbB-2 localization upon R2 treatment sensitized MDA-453 cells to trastuzumab antiproliferative effects. These findings support that the combination of R2 with trastuzumab may offer clinical benefits to TNBC and, eventually, to ErbB-2-positive BC patients bearing NWTErbB-2-positive tumors. The translational relevance of R2 was highlighted by TNBC growth abrogation in multiple preclinical models, including tumor explants and xenografts. We acknowledge that the translation of this experimental R2 therapy into the clinical setting is a challenge. However, in light of the lack of efficient targeted therapies for TNBC, innovative and alternative approaches are required. R2 doses in our in vivo studies were comparable to those of other small molecules such as neratinib, alpelisib, or everolimus, already approved for BC treatment [50, 51]. R2 specificity for inhibiting growth of NErbB-2-positive TNBC and its lack of in vivo toxicity offers the unique opportunity to repurpose R2 as an anticancer agent.

We were the first to identify the nuclear presence of WTErbB-2 and ErbB-2c in TNBC cells and further revealed NErbB-2 as a marker of poor prognosis in TNBC [9]. Our current discoveries identify R2 as a tool to target NErbB-2 retrograde transport. This novel theragnostic approach could greatly improve the outcome of TNBC patients with NErbB-2-positive tumors.

## Materials and methods

### Reagents and antibodies

Retro-2 (2-([(5-Methyl-2-thienyl)methylene]amino)-N-phenylbenzamide; C_19_H_16_N_2_OS; R2) was purchased from EMD Millipore (Temecula, CA, USA) and stored at 50 mg/mL in DMSO at −20 °C. R2 spontaneously cyclizes to Retro-2^cycl^ [52], the mixture is referred to as R2 herein. The purified Retro-2.1 (6-Fluoro-1-methyl-2-(5-(2-methylthiazol-4-yl)thiophen-2-yl)-3-phenyl-2,3-dihydroquinazolin-4(1H)-one; C_23_H_18_FN_3_OS_2_; R2.1) *S-*enantiomer (*(S)-*R2.1) and *R*-enantiomer (*(R)-*R2.1) were purchased from ChiroBlock (Wolfen, Germany). Heregulin β1 (HRGβ1) was purchased from R&D Systems Inc (Minneapolis, MN, USA). Medroxyprogesterone acetate (MPA) and Dulbecco’s modified Eagle’s medium: Ham’s F12 1:1 (DMEM) were purchased from Sigma Aldrich (Saint Louis, USA). RPMI 1640 was from Gibco (Thermo Fisher Scientific, CA, USA), trastuzumab was from Hoffmann-La Roche Ltd Genentech Inc. (Basel, Switzerland), and dimethyl sulfoxide (DMSO) was from Merck (USA). The antibodies are listed under Table S2.

### Cell lines, treatments, and proliferation assays

MDA-468, MDA-231, 4T1, BT-474, T47D, MCF-7, and MCF10A cells were obtained from the American Type Culture Collection (Manassas, VA, USA) and were maintained according to the supplier’s instructions. MDA-453 cells were a gift from DJ Slamon (UCLA, Los Angeles, CA, USA) and were cultured as described [7, 53]. Cell lines were authenticated by short tandem repeat DNA profiling and were regularly screened for mycoplasma contamination. Primary cultures of epithelial cells from C4HD tumors induced by MPA in mice were performed as described [29]. Cells were treated with R2 (20–100 µM), *(S)-*R2.1 and *(R)-*R2.1 (5–100 µM), or control vehicle (0.1–0.6% DMSO depending on R2 concentration). All experiments were done in complete growth media (DMEM or RPMI 1640 supplemented with 10% fetal calf serum, FCS), except in experiments assessing the effects of HRGβ1 or MPA on ErbB-2 migration or proliferation. Prior to stimulation with HRGβ1 (40 ng/ml) or MPA (10 nM), cells were starved for 48 h in DMEM 0.1% charcoalized FCS for T47D and C4HD cells, or for 24 h in DMEM 1% chFCS for BT-474. Cell proliferation was evaluated by incorporation of 1 μCi [^3^H]-thymidine during the last 18 h of incubation (New England Nuclear, DuPont; specific activity 20 Ci/mmol) or by cell count, as previously described [7, 9]. Assays were performed in octuplicate. In experiments assessing R2 effects on HRGβ1-stimulated proliferation and for trastuzumab assays, cells were pretreated with R2 for 90 min and then treated with HRGβ1 or trastuzumab (10 µg/mL), or remained untreated for 24 h.

### Western blot (WB) and subcellular fractionation

Lysates were prepared from cells subjected to different treatments and analyzed by SDS-PAGE and WB as described [7, 9]. For experiments assessing the expression of the different ErbB-2 isoforms, 50 μg of protein lysates were resolved by SDS-PAGE on 6% gels [9]. Experiments analyzing phosphorylation levels of ErbB-2, Akt, and Erk1/2 were performed in parallel with confocal microscopy studies showing R2 effects on NErbB-2 localization. Signal intensities of phospho-ErbB-2 (pErbB-2), pAkt, and pErk1/2 bands were analyzed by densitometry using ImageJ software (National Institutes of Health), and normalized to total protein bands. Similarly, signal intensities of ErbB-2, cyclin D1, and Erk5 bands were normalized to β tubulin bands, used as loading control. The subcellular fractionation protocol was based on that from Dr. Richard Patten at Abcam (https://www.abcam.com/protocols/subcellular-fractionation-protocol) and was performed as previously described [9]. In experiments assessing subcellular fractionation of ErbB-2, AR, and c-Jun, total protein bands were normalized to β tubulin and histone H3 bands, which were used as loading control and for fractionation efficiency.

### Immunofluorescence (IF) and confocal microscopy

Techniques were performed as described [7, 9]. For IF and confocal microscopy in cell cultures, ErbB-2, Stat3, E-cadherin, EEA1, calnexin, and GM130 were localized using the antibodies described in Table S2, followed by incubation with AF-conjugated secondary antibodies. Negative controls were carried out using PBS instead of primary antibodies or 5X competitive peptide (sc-284 P, Santa Cruz Biotechnology) when ErbB-2 C-18 was used. Nuclei were detected either by propidium iodide staining (PI, 5 μg/ml, Invitrogen) or DAPI (4′,6-diamidino-2-phenylindole). IF signal was detected using an Olympus FV1000 confocal laser scanning microscope. Confocal images were obtained using identical acquisition parameters. Quantitative analysis of ErbB-2 subcellular localization was performed with ImageJ software as reported [9]. For a detailed description of quantitative analysis of ErbB-2 confocal images and of Manders’ colocalization analysis, see Supplementary Materials and Methods. ErbB-2 IF analysis of paraffin-embedded tissue samples was performed as described [54] and details are provided in Supplementary Materials and Methods.

### Flow cytometry analysis

Cell-cycle distribution and trastuzumab binding were evaluated by flow cytometry as described [7, 55, 56]. Apoptosis and necrosis were investigated through cell-surface binding of fluorescent annexin V by using the Annexin-V binding assay (Biolegend, San Diego, CA, USA) [57] and 7-aminoactinomycin (7-AAD) staining, following the manufacturer’s instructions. Cell cycle, apoptosis, and trastuzumab-binding analyses were performed using a Canto II flow cytometer (Becton-Dickinson, La Jolla, CA, USA). The acquired data were analyzed with FlowJo software. For trastuzumab-binding analyses, background staining was evaluated in cells incubated with an isotype control human IgG followed by anti-human AlexaFluor-647-conjugated antibody. Delta mean fluorescence intensity (MFI) values were obtained by subtracting the MFI of cells incubated with control antibody from the MFI of trastuzumab-incubated cells.

### Plasmids and transient transfections

The green fluorescence protein (GFP)-tagged human ErbB-2 mutant, which lacks the putative nuclear localization signal (NLS) (aa 676-KRRQQKIRKYTMRR-689; hErbB-2ΔNLS) [13], was generously provided by Dr. MC Hung (University of Texas, M.D. Anderson Cancer Center, Houston, TX, USA). The expression plasmid encoding GalNAc-T-Cherry, a Golgi complex marker [58], was kindly gifted by Dr. Daniotti (CIQUIBIC-CONICET). The empty vector pEGFP-N1 was obtained from BD Biosciences-Clontech. Site-directed mutagenesis was performed on the WTErbB-2-pEYFP to produce the deletion of a region containing leucines 11–13 (ErbB-2∆11–13-pEYFP, ErbB-2∆11–13), using the following primers: 5′-GGCAAGAGGGCCCCCCAGCGGC-3′ and 5′-GCCGCTGGGGGGCCCTCTTGCC-3′. Successful mutation of the sequence was verified by DNA sequencing at Macrogen (Seoul, SK). Cells were transfected for 72 h with 2 µg of expression vectors using X-tremeGENE HP (Roche) following the manufacturer’s instructions. In the case of the GalNAc-T-Cherry plasmid, cells were transfected for 48 h before treatment with R2. Transfection efficiencies, evaluated using the pEGFP-N1 vector and determined by the percentage of cells exhibiting GFP 96 h after transfection, varied between 60 and 70%.

### RNA isolation and RT-qPCR

RNA was obtained and mRNA levels were detected by RT-qPCR as described [7, 9]. The following primers were used [7, 9]: for GAPDH cDNA, 5′-CAGTCAGCCGCATCTTCTTTTG-3′ and 5′-ACCAGAGTTAAAAGCAGCCCT-3′; for cyclin D1 cDNA, 5′-TATTGCGCTGCTACCGTTGA-3′ and 5′-CCAATAGCAGCAAACAATGTGAAA-3′; for Erk5 cDNA, 5′-CACACCGCTGCCTCTGTAG-3′ and 5′-TGCCTATGGTCTCGATGATCT-3′. The fold change of mRNA expression was calculated by normalizing the absolute mRNA amounts to GAPDH mRNA levels, used as an internal control, and by setting the value of control cells to 1.

### Preclinical models

All animal studies were conducted in accordance with the highest standards of animal care as outlined by the NIH Guide for the Care and Use of Laboratory Animals, and were approved by the IBYME Animal Research Committee. MDA-468 and MDA-231 tumor xenografts were established in two-month-old female NIH(S)-nude mice (La Plata National University, Argentina) as previously described [9]. When tumors reached 50 mm^3^ (ref. [9]), mice were randomly assigned to treatment groups (*n* = 6) and injected intraperitoneally with 500 µl of sterile saline solution supplemented with 10% DMSO alone (vehicle) or R2 (20 mg/kg) twice weekly [17]. Tumor volumes, growth rates, and percentage of growth inhibition were calculated as described [9, 57]. At necropsy, tumors and organs were extracted, formalin-fixed, and paraffin-embedded for further analyses with hematoxylin and eosin (H&E) staining (see Supplementary Materials and Methods) or IF detection.

Ex vivo culture of tumor explants was performed as described [28]. For murine explant culture, 4T1 cells (1 × 10^4^) were first injected subcutaneously in two-month-old female BALB/c mice and allowed to grow, until they reached 50 mm^3^. Tumors were then isolated under sterile conditions, collected in DMEM–10% FCS, and subjected to mechanical breakdown by dissection using a surgical blade [59]. Sample sections of the 4T1 tumors (1-mm^3^ thick) were cut and placed into neutral-buffered formalin for paraffin embedding (day-0 sample) or in quadruplicate on presoaked gelatin sponge with 500 μL DMEM–10% FCS inside 24-well plates [28, 59] for further treatment with R2 (100 µM) or vehicle. For MDA-468 ex vivo culture, MDA-468 tumor xenografts were established as indicated. When tumors reached 50 mm^3^, they were resected under sterile conditions and subjected to R2 treatment or transfected with hErbB-2ΔNLS or pEGFP-N1 vectors. At the end of the experiments, 4T1 and MDA-468 explants were formalin-fixed and paraffin-embedded for H&E and IF analyses, or preserved in RNAlater (Qiagen, Hilden, Germany) for RNA isolation (in MDA-468 explants). For assessment of 5-ethynyl-2′-deoxyuridine (EdU) incorporation, EdU (10 µM) was added to the culture medium 24 h prior to harvest [59, 60]. Cellular uptake of EdU in formalin-fixed and paraffin-embedded explants was stained by Click-iT EdU Imaging Kits (Thermo Fisher Scientific) following the manufacturer’s instruction.

### Bioinformatics

A detailed description of in silico analyses is provided in Supplementary Materials and Methods.

### Statistical analysis

Analyses were performed using Prism 8 (GraphPad Software) and STATA version 15 (Stata Corp. LLC). Information on biological replicates and statistical significance are reported in the respective figure legends. When two groups were compared, the two-tailed Student’s t-test was used. When three or more groups were compared, the one-way ANOVA with Tukey’s multiple comparison test was used. Homoscedasticity of the variances was analyzed in every case. Two-way ANOVA with repeated measures followed by Bonferroni post test was applied to assess statistical significance of differences in tumor growth kinetics among groups as previously described [61]. *P-*values <0.05 were regarded as statistically significant.

## Supplementary information

Supplementary Materials and Methods

Table S1

Table S2

Figure S1

Figure S2

Figure S3

Figure S4

Figure S5

Figure S6

Figure S7

Figure S8

Figure S9

Reproducibility Checklist

## Data Availability

All data generated or analyzed during this study are included in this article (and its Supplementary information files).
