# Correction: Halting ErbB-2 isoforms retrograde transport to the nucleus as a new theragnostic approach for triple-negative breast cancer

**DOI:** 10.1038/s41419-023-06339-1

**Published:** 2023-12-15

**Authors:** Santiago Madera, Franco Izzo, María F. Chervo, Agustina Dupont, Violeta A. Chiauzzi, Sofia Bruni, Ezequiel Petrillo, Sharon S. Merin, Mara De Martino, Diego Montero, Claudio Levit, Gabriel Lebersztein, Fabiana Anfuso, Agustina Roldán Deamicis, María F. Mercogliano, Cecilia J. Proietti, Roxana Schillaci, Patricia V. Elizalde, Rosalía I. Cordo Russo

**Affiliations:** 1 Laboratory of Molecular Mechanisms of Carcinogenesis and Molecular Endocrinology, Instituto de Biología y Medicina Experimental (IBYME), CONICET, Vuelta de Obligado 2490, C1428ADN Buenos Aires, Argentina; 2 New York Genome Center, New York, NY USA; 3 Meyer Cancer Center, Weill Cornell Medicine, New York, NY USA; 4 Universidad de Buenos Aires (UBA), Facultad de Ciencias Exactas y Naturales, Departamento de Fisiología, Biología Molecular y Celular and CONICET-UBA, Instituto de Fisiología, Biología Molecular y Neurociencias (IFIBYNE), C1428EHA Buenos Aires, Argentina; 5 Department of Radiation Oncology, Weill Cornell Medicine, New York, NY USA; 6 Servicio de Ginecología, Sanatorio Sagrado Corazón, Buenos Aires, Argentina

**Keywords:** Breast cancer, Protein translocation, Oncogenes, Nuclear transport, Targeted therapies

Correction to: *Cell Death and Disease* 10.1038/s41419-022-04855-0, published online 09 May 2022

The original version of the manuscript contained an error in Fig. 1H and its corresponding Source data (Fig. S9C). Specifically, the β tubulin and Histone H3 blots corresponding to MDA-231 cells in the bottom panel of Fig. 1H were unintentionally mistaken while assembling the figures. We have rectified this issue by including the correct blots for β tubulin and Histone H3 controls in MDA-231 cells within the corrected Fig. 1H. Additionally, the corrected Source data is available in the corrected Fig. S9C. This error in the loading control blots does not impact in the interpretation of the results presented in Fig. 1H, nor does it affect the general conclusions of Fig. 1 or of the entire article. The authors sincerely apologize for any confusion the error may have caused. The corrected figures can be found below. The original article has been corrected.

**Fig. 1:**
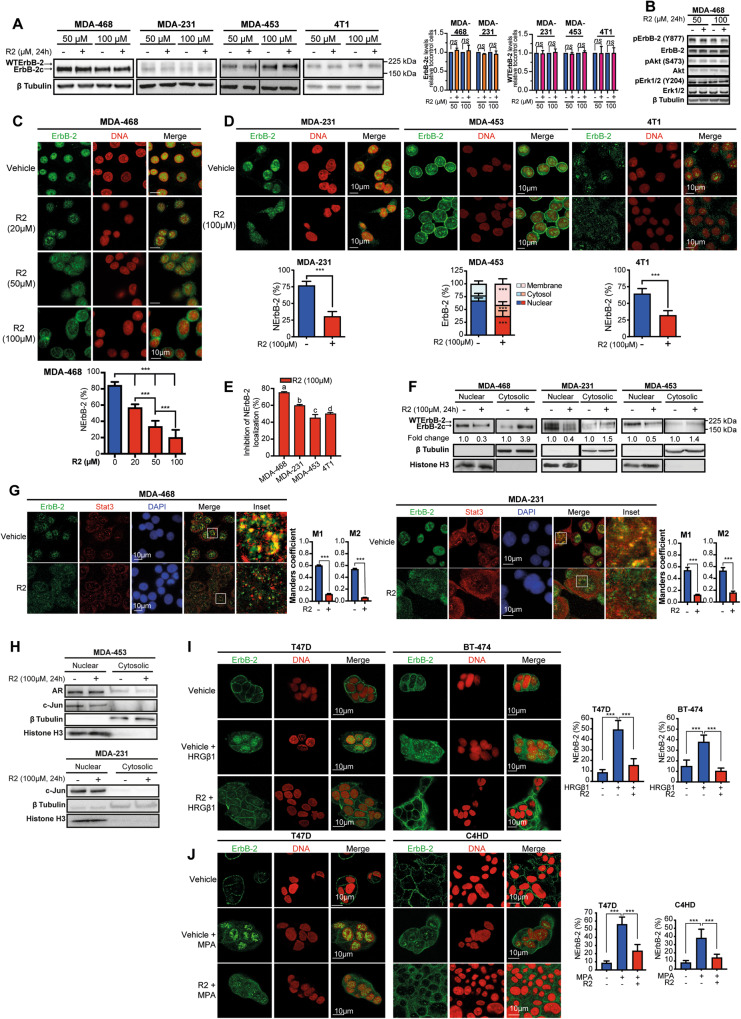
R2 evicts WTErbB-2 and ErbB-2c from the nucleus of BC cells. **A** Left: Representative WB of ErbB-2 expression. Right: signal intensities of ErbB-2c and WTErbB-2 were analyzed by densitometry from three independent WBs performed as indicated. Fold change was calculated by normalizing the absolute levels of each ErbB-2 isoform to those of β tubulin, setting the value of vehicle-treated cells to 1. **B** Representative WB of cell lysates with the indicated phosphospecific or total antibodies. **C**, **D** ErbB-2 immunofluorescence (IF) in cells treated with R2 or vehicle (24 h). Bottom panels: quantitative analysis of ErbB-2 subcellular localization. Fluorescence intensity of nuclear, cytosolic, and membrane ErbB-2 was quantified and is plotted as percentage (mean ± SD, *n* = 50 per group) relative to total ErbB-2 in each cell. **E** Inhibition of NErbB-2 localization in cells from **C**, **D** (mean ± SEM). For **C**, **D** vs **A**: *P* < 0.001; for **C**, **A** vs **B**: *P* < 0.01, for **D** vs **B**: *P* < 0.05. **F** Nuclear and cytosolic lysates were analyzed by WB. Fold change was calculated for each compartment by normalizing ErbB-2 levels in treated vs control cells (value set to 1). **G** ErbB-2 and Stat3 were localized by IF and confocal microscopy in cells treated as in (**D**). Merged images show colocalization in yellow. The insets show boxed areas in detail. Right: Quantitative analysis of colocalization with Manders’ coefficients (M1 and M2, mean ± SEM, *n* = 50 per group). **H** Subcellular distributions of AR and c-Jun evaluated as in (**F**). **I**, **J** Cells were pretreated with R2 (100 µM) or vehicle for 24 h and then treated with HRGβ1 (60 min) (**I**) or MPA (90 min) (**J**). ErbB-2 localization and NErbB-2 levels are depicted as in (**C**). ns: not significant, ***P < 0.001. For (**A**–**J**), *n* = 3. Original uncropped WB images are shown in Fig. S9.

The original article has been corrected.

### Supplementary information

Corrected_Supplementary_Figure 9

