# Supplementary material for: Halting ErbB-2 isoforms retrograde transport to the nucleus as a new theragnostic approach for triple-negative breast cancer
Source: Cell Death Dis. 2022 May 9;13(5):447. doi: 10.1038/s41419-022-04855-0 (PMC9084267; doi:10.1038/s41419-022-04855-0)
Supplement: Supplementary file 1 — Supplementary Materials and Methods [file 41419_2022_4855_MOESM1_ESM.docx]

**SUPPLEMENTARY DATA**

Halting ErbB-2 Isoforms Retrograde Transport to the Nucleus as a New Theragnostic Approach for Triple Negative Breast Cancer

Santiago Madera^1^, Franco Izzo^2,3^, María F. Chervo^1^, Agustina Dupont^1^, Violeta A. Chiauzzi^1^, Sofia Bruni^1^, Ezequiel Petrillo^4^, Sharon S. Merin^1^, Mara De Martino^5^, Diego Montero^1^, Claudio Levit^6^, Gabriel Lebersztein^6^, Fabiana Anfuso^6^, Agustina Roldán Deamicis^1^, María F. Mercogliano^1^, Cecilia J. Proietti^1^, Roxana Schillaci^1^, Patricia V. Elizalde^1§^, Rosalía I. Cordo Russo^1§^

**Supplementary Materials and Methods**

**Figure S1, related to Figure 1**

**Figure S2, related to Figure 1**

**Figure S3, related to Figure 1**

**Figure S4, related to Figure 3**

**Figure S5, related to Figure 4**

**Figure S6, related to Figure 6**

**Figure S7, related to Figure 6**

**Figure S8, related to Figure 6**

**Figure S9, related to Figure 1**

**Table S1, related to Figure 4**

**Table S2, related to Materials and Methods section**

**Supplementary Materials and Methods**

**Immunofluorescence (IF) and confocal microscopy**

Quantitative analysis of ErbB-2 subcellular localization was performed with ImageJ software as reported [1]. Briefly, we performed quantitative analysis of confocal images to evaluate the percentages of ErbB-2 localized at the nucleus, the cytosol, and the plasma membrane (depending on the cell line). Segmentation of the whole cell was performed manually using ErbB-2 images. The plasma membrane was defined as the difference between the image of the cell and a binary erosion (iterations: 5-25), and the nuclear compartment was defined according to the nuclear stain (PI or DAPI). The integrated fluorescence intensity value (mean fluorescence intensity per unit area) was obtained for total ErbB-2 (TErbB-2), and ErbB-2 at the membrane (MErbB-2), cytosol (CErbB-2), or nucleus (NErbB-2). Fluorescence background (median) was subtracted in all cases. To compute ErbB-2 subcellular distribution, the ratio of integrated fluorescence intensity was calculated for at least 50 cells (MErbB-2/TErbB2; CErbB-2/TErbB-2; NErbB-2/TErbB-2).

For colocalization analysis, Manders’ colocalization coefficients (M1, M2; refs. 2, 3) were calculated for the proportion of the overlapping signal of channels in each condition. All quantitative microscopy measurements were performed in individual cells (n = 50 for each condition in Fig. 1G and n = 30 for each condition in Fig. 5C-F and in Fig. 6F-G). FIJI software (ImageJ, NIH) plugin Coloc2 was used for quantification as described [3, 4] and image processing was applied only for presentation. Unpaired t-test analysis was performed to compare the means of channels. Visualization of signal intensities was done using line intensity profiles in FIJI, as described [3]. For each condition, the corresponding intensity profiles along the yellow line in the inset are shown. The line profiles from the two different channels were merged into one graph. Signal intensities (absolute gray values from a 16-bit raw image) are plotted on the graph, where Y axis is the signal intensity and X axis is the line length along the cell.

ErbB-2 IF analysis of paraffin-embedded tissue samples was performed as we described [5]. Briefly, antigen retrieval was performed by immersing the sections in 10 mM sodium citrate buffer pH 6 and microwaving at high power for 4 min. Slides were blocked in Modified Hank’s Buffer (MHB) with 5% BSA for 30 min and incubated overnight at 4ºC with the ErbB-2 C-18 antibody. Slides were then incubated with an anti-rabbit IgG-AF-488 antibody. Reduction of the autofluorescent background was performed by incubation with Sudan Black B 0.1% (Sigma-Aldrich). Nuclei were stained with DAPI. Slides were analyzed by an Olympus FV1000 confocal laser microscopy system. Negative controls were carried out with MHB instead of primary antibodies. C4HD tumors from the model of mammary tumors induced by progestins were used as positive controls [6, 7].

**Bioinformatics**

Human and murine ErbB-2 protein sequences from RefSeq database (release 204) were used to conduct multiple sequence alignment with the Clustal Omega online tool [8] at European Bioinformatics Institute (EBI) (https://www.ebi.ac.uk/Tools/msa/clustalo/). Default parameters were used for the analysis.

For analysis of Importin β1 mRNA expression in BC cell lines, we assessed the Cancer Cell Line Encyclopedia (CCLE) dataset [9]. To classify BC cell lines, literature mining was performed for estrogen receptor, progesterone receptor, and ErbB-2 status and cells were classified into ErbB-2-positive (ErbB-2+) or TN, as previously reported [10]. ErbB-2+ BC cell lines include AU565, HCC1569, HCC1954, JIMT1, SKBR3, UACC893, BT474, EFM192A, HCC1419, HCC202, HCC2157, HCC2218, MDA-361, UACC812, and ZR7530 cell lines; TNBC cell lines include BT20, CAL120, CAL148, CAL51, CAL851, DU4475, HCC1143, HCC1187, HCC1395, HCC1500, HCC1599, HCC1806, HCC1937, HCC38, HDQP1, HMC18, Hs578T, MDA-156, MDA-436, and the following four models: HCC70, MDA-468, MDA-231 and MDA-453 which are NErbB-2+ TNBC models (ref. [1], this work). Processed mRNA expression data from the CCLE was downloaded from the CCLE portal (https://portals.broadinstitute.org/ccle). Two-sided Pearson’s correlation test was used to evaluate statistical significance in all these analyses.

The BLAST algorithm (http://www.ncbi.nlm.nih.gov/blast) was used to analyze 5’UTR, 3’UTR, and CCDSs regions of human ErbB-2 transcript T1 and T3 as defined by NCBI (NM_004448.4 and NM_001289936.2, respectively, Version Nov 10 2020).

The RNAfold web server (http://rna.tbi.univie.ac.at/) from the University of Vienna was used [11] as a predictive tool for the secondary structure folding of ErbB-2 mRNA sequences. The full length mRNA sequences of Transcript 1 and Transcript 3 of ErbB-2 were used [1]. Both minimum free energy (MFE) and the newer centroid prediction models were used. A stable RNA secondary structure with the lowest energy state is predicted by the MFE model [11, 12] whilst the algorithm for the centroid model calculates the statistically most likely Boltzmann weighted ensemble of predicted secondary structures [13].

Classical NES were identified with NetNES 1.1 software [14] using the default parameters. The NetNES 1.1 server predicts leucine-rich nuclear export signals (NES) in eukaryotic proteins using a combination of neural networks and hidden Markov models.

**Histopathological analysis**
Hematoxylin and eosin (H&E) staining was performed on 5 μm slide sections of MDA-468 and MDA-231 tumors, or of 4T1 and MDA-468 explants, and used for histopathological examination as we previously described [1, 10]. Mitotic figure counts were performed in 10 consecutive high power fields (HPF, 400X magnification) using a Leica DM500 light microscope (0.45 mm diameter of the HPF). The identification of well-defined mitotic figures was performed as described [1]. In brief, the most poorly differentiated peripheral tumor area was used for counting mitoses; necrotic, heavily inflamed, or benign areas were avoided. This area, defined as the measurement area, was minimally 1x1 mm and maximally 5x5 mm. In the measurement area, at 400X magnification (objective 40, field diameter 450 μm at the specimen level), mitoses were counted in 10 consecutive neighboring fields of vision with the highest number of cells in the most cellular area. Only certain mitoses were counted, doubtful structures and apoptotic bodies were ignored. Percentage of tumor necrosis was evaluated at 40X magnification using a Leica DM500 light microscope. Necrotic areas were characterized by cell and nuclear swelling, pale eosinophilic cytoplasm, nuclear dissolution (karyolysis), nuclear fragments (karyorrhexis) and loss of cellular detail with shadows of tumor cells visible to variable extent. Some degree of nuclear condensation (pyknosis) may be present. Adjacent cellular debris and inflammation (neutrophils, macrophages, etc.) may also be present if cell membrane leakage or rupture occurred.

**Immunohistochemistry**

For immunohistochemistry (IHC), tissue sections of MDA-468 explants were processed as previously described [10, 15] and incubated with an antibody against GFP (see Table S2). Negative controls were carried out using an isotype-nonspecific IgG antibody instead of the first antibody.

**Supplementary References**

1. Chervo MF, Cordo Russo RI, Petrillo E, Izzo F, De Martino M, Bellora N, et al. Canonical ErbB-2 isoform and ErbB-2 variant c located in the nucleus drive triple negative breast cancer growth. Oncogene. 2020;39(39):6245-62.

2. Manders EMM, Verbeek FJ, Aten JA. Measurement of co-localization of objects in dual-colour confocal images. Journal of microscopy. 1993;169(3):375-82.

3. Rajani S, Gell C, Abakir A, Markus R. Computational Analysis of DNA Modifications in Confocal Images. Methods in molecular biology. 2021;2198:227-54.

4. Dunn KW, Kamocka MM, McDonald JH. A practical guide to evaluating colocalization in biological microscopy. American journal of physiology Cell physiology. 2011;300(4):C723-42.

5. Schillaci R, Guzman P, Cayrol F, Beguelin W, Diaz Flaque MC, Proietti CJ, et al. Clinical relevance of ErbB-2/HER2 nuclear expression in breast cancer. BMCCancer. 2012;12(1):74.

6. Beguelin W, Diaz Flaque MC, Proietti CJ, Cayrol F, Rivas MA, Tkach M, et al. Progesterone receptor induces ErbB-2 nuclear translocation to promote breast cancer growth via a novel transcriptional effect: ErbB-2 function as a coactivator of Stat3. MolCell Biol. 2010;30(23):5456-72.

7. Balana ME, Lupu R, Labriola L, Charreau EH, Elizalde PV. Interactions between progestins and heregulin (HRG) signaling pathways: HRG acts as mediator of progestins proliferative effects in mouse mammary adenocarcinomas. Oncogene. 1999;18(46):6370-9.

8. Sievers F, Wilm A, Dineen D, Gibson TJ, Karplus K, Li W, et al. Fast, scalable generation of high-quality protein multiple sequence alignments using Clustal Omega. Molecular systems biology. 2011;7:539.

9. Ghandi M, Huang FW, Jane-Valbuena J, Kryukov GV, Lo CC, McDonald ER, III, et al. Next-generation characterization of the Cancer Cell Line Encyclopedia. Nature. 2019;569(7757):503-8.

10. Madera S, Chervo MF, Chiauzzi VA, Pereyra MG, Venturutti L, Izzo F, et al. Nuclear PDCD4 Expression Defines a Subset of Luminal B-Like Breast Cancers with Good Prognosis. Hormones & cancer. 2020;11(5-6):218-39.

11. Lorenz R, Bernhart SH, Honer Zu Siederdissen C, Tafer H, Flamm C, Stadler PF, et al. ViennaRNA Package 2.0. Algorithms for molecular biology : AMB. 2011;6:26.

12. Zuker M, Stiegler P. Optimal computer folding of large RNA sequences using thermodynamics and auxiliary information. Nucleic acids research. 1981;9(1):133-48.

13. Ding Y, Chan CY, Lawrence CE. RNA secondary structure prediction by centroids in a Boltzmann weighted ensemble. Rna. 2005;11(8):1157-66.

14. la Cour T, Kiemer L, Molgaard A, Gupta R, Skriver K, Brunak S. Analysis and prediction of leucine-rich nuclear export signals. Protein engineering, design & selection : PEDS. 2004;17(6):527-36.

15. Mercogliano MF, De Martino M, Venturutti L, Rivas MA, Proietti CJ, Inurrigarro G, et al. TNFalpha-Induced Mucin 4 Expression Elicits Trastuzumab Resistance in HER2-Positive Breast Cancer. Clin Cancer Res. 2017;23(3):636-48.

16. Katoh M, Katoh M. Identification and characterisation of mouse Erbb2 gene in silico. International journal of oncology. 2003;23:831-5.

17. Iwarsson K, Rehbinder C. A study of different euthanasia techniques in guinea pigs, rats, and mice. Animal response and postmortem findings. Scand J Lab Anim Sci. 1993;20:191-205
