# Supplementary figures and images for: Halting ErbB-2 isoforms retrograde transport to the nucleus as a new theragnostic approach for triple-negative breast cancer
Source: Cell Death Dis. 2022 May 9;13(5):447. doi: 10.1038/s41419-022-04855-0 (PMC9084267; doi:10.1038/s41419-022-04855-0)

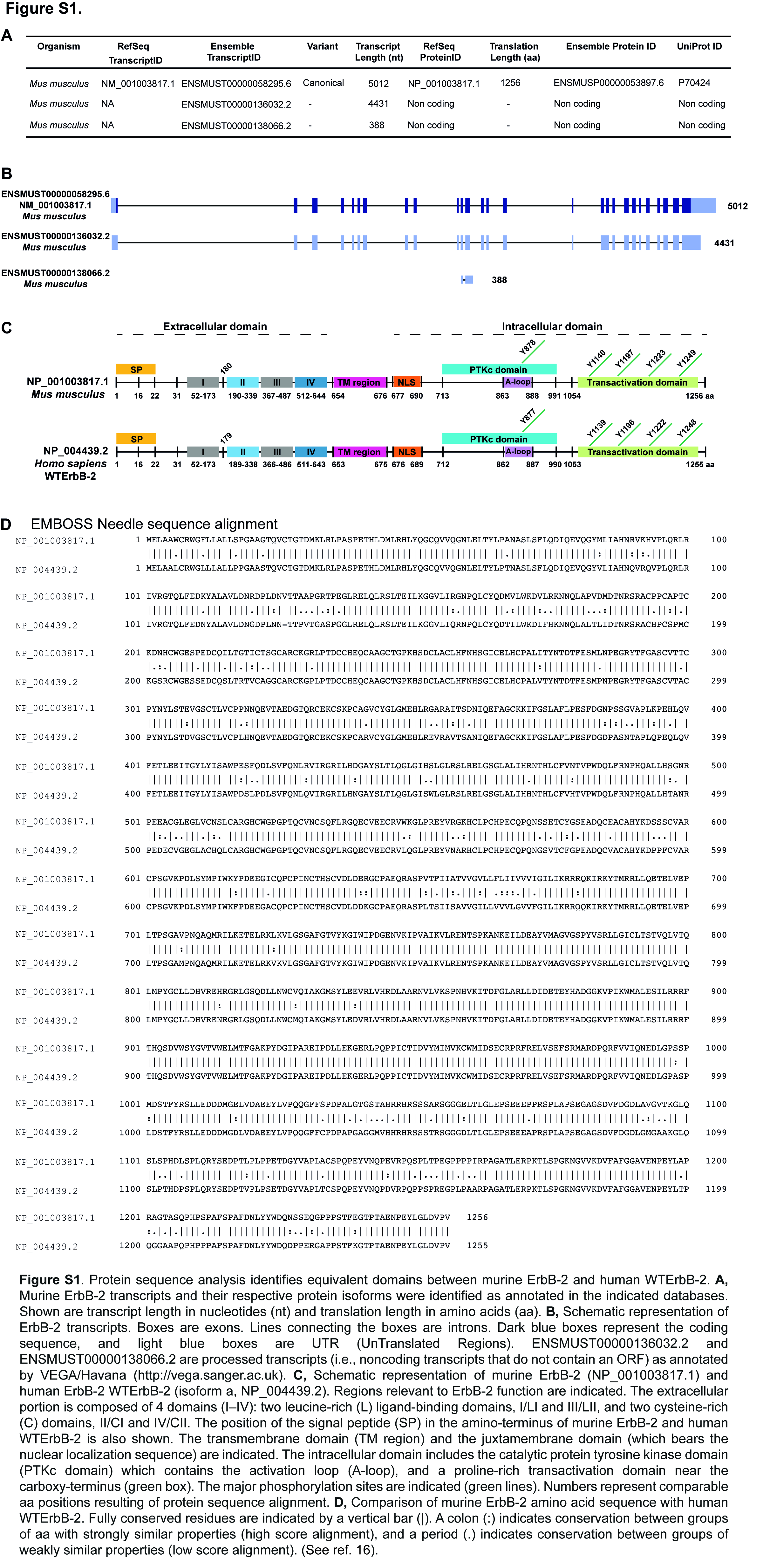

Supplement: Supplementary file 4 — Figure S1 [file 41419_2022_4855_MOESM4_ESM.tif]

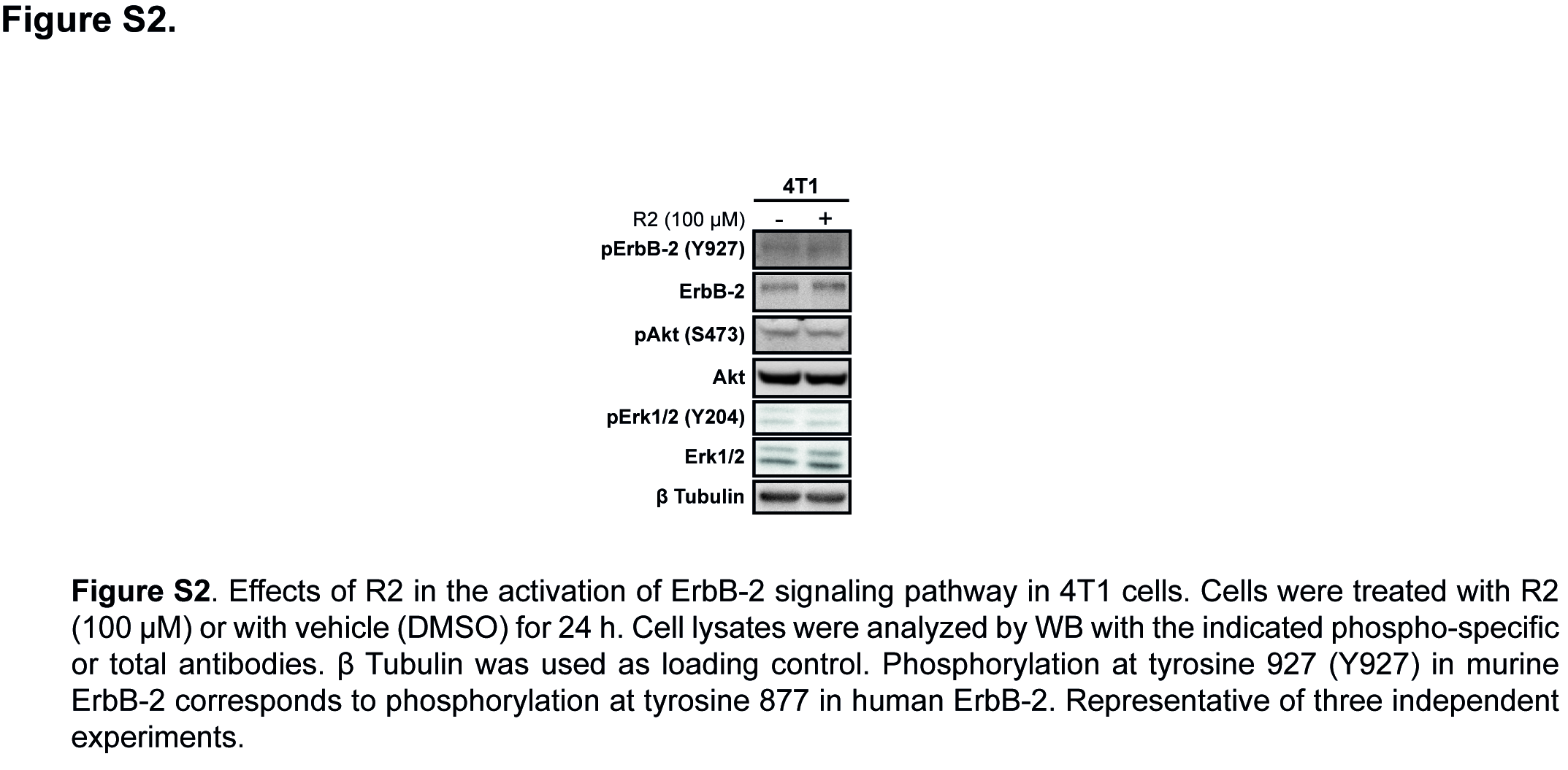

Supplement: Supplementary file 5 — Figure S2 [file 41419_2022_4855_MOESM5_ESM.tif]

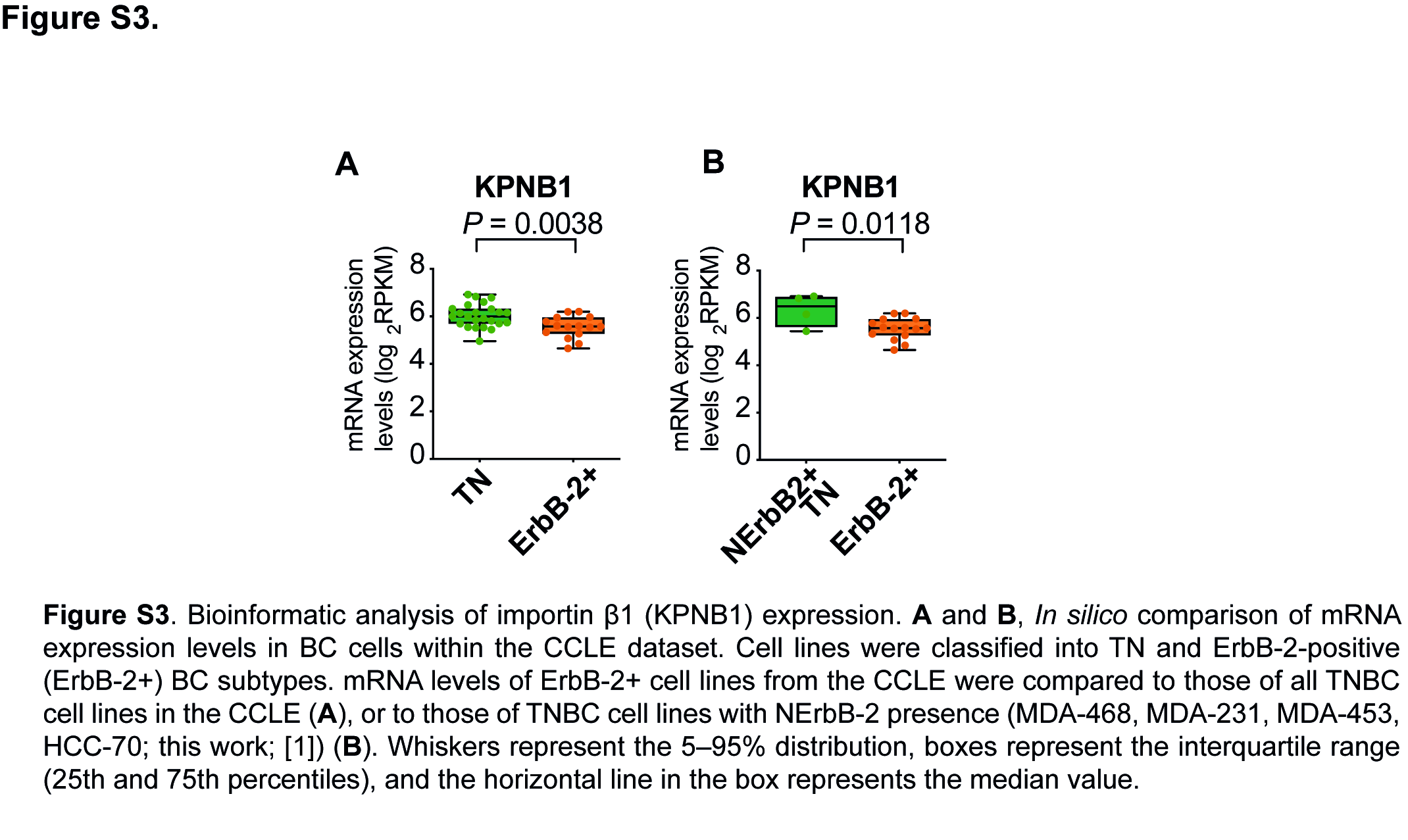

Supplement: Supplementary file 6 — Figure S3 [file 41419_2022_4855_MOESM6_ESM.tif]

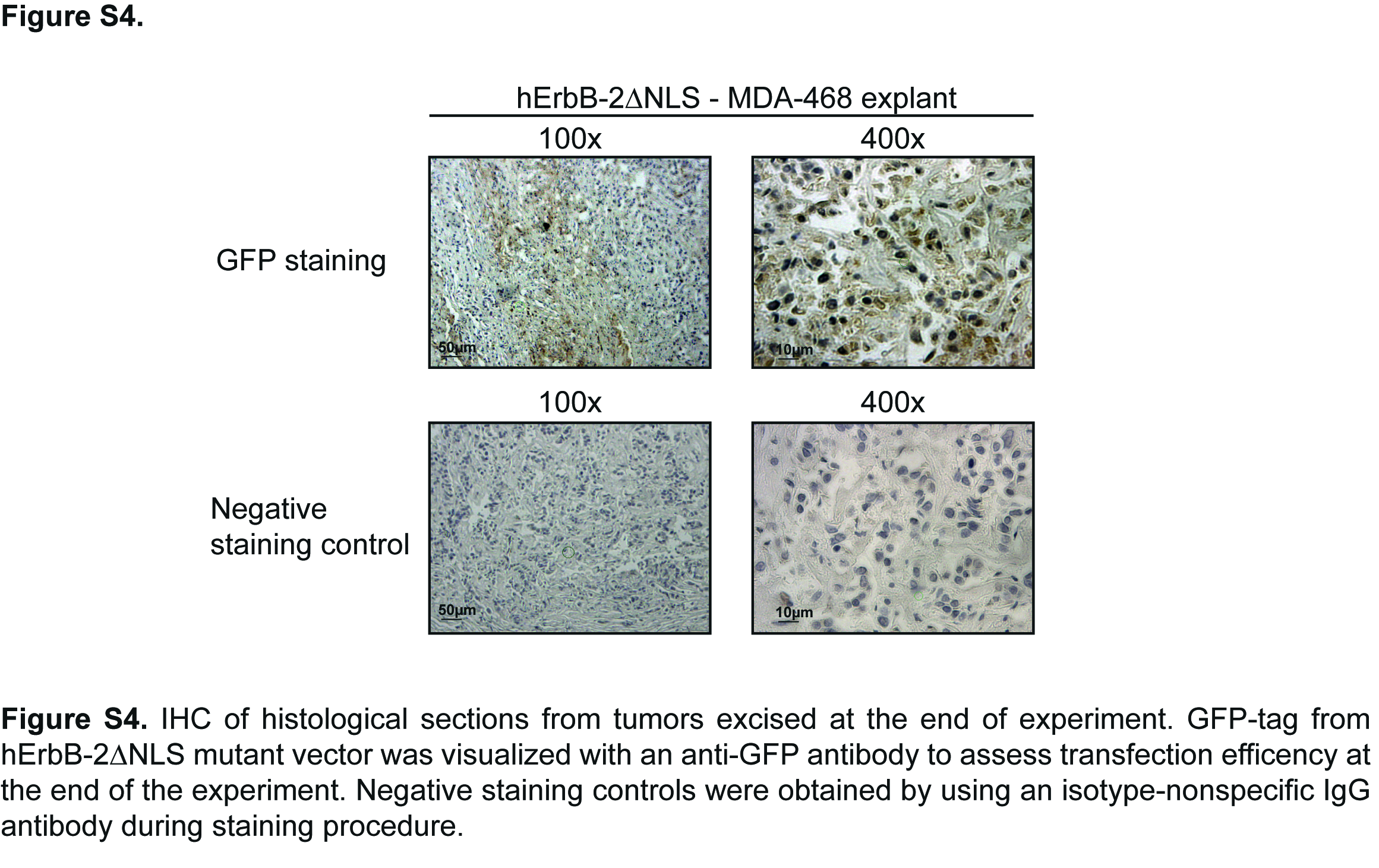

Supplement: Supplementary file 7 — Figure S4 [file 41419_2022_4855_MOESM7_ESM.tif]

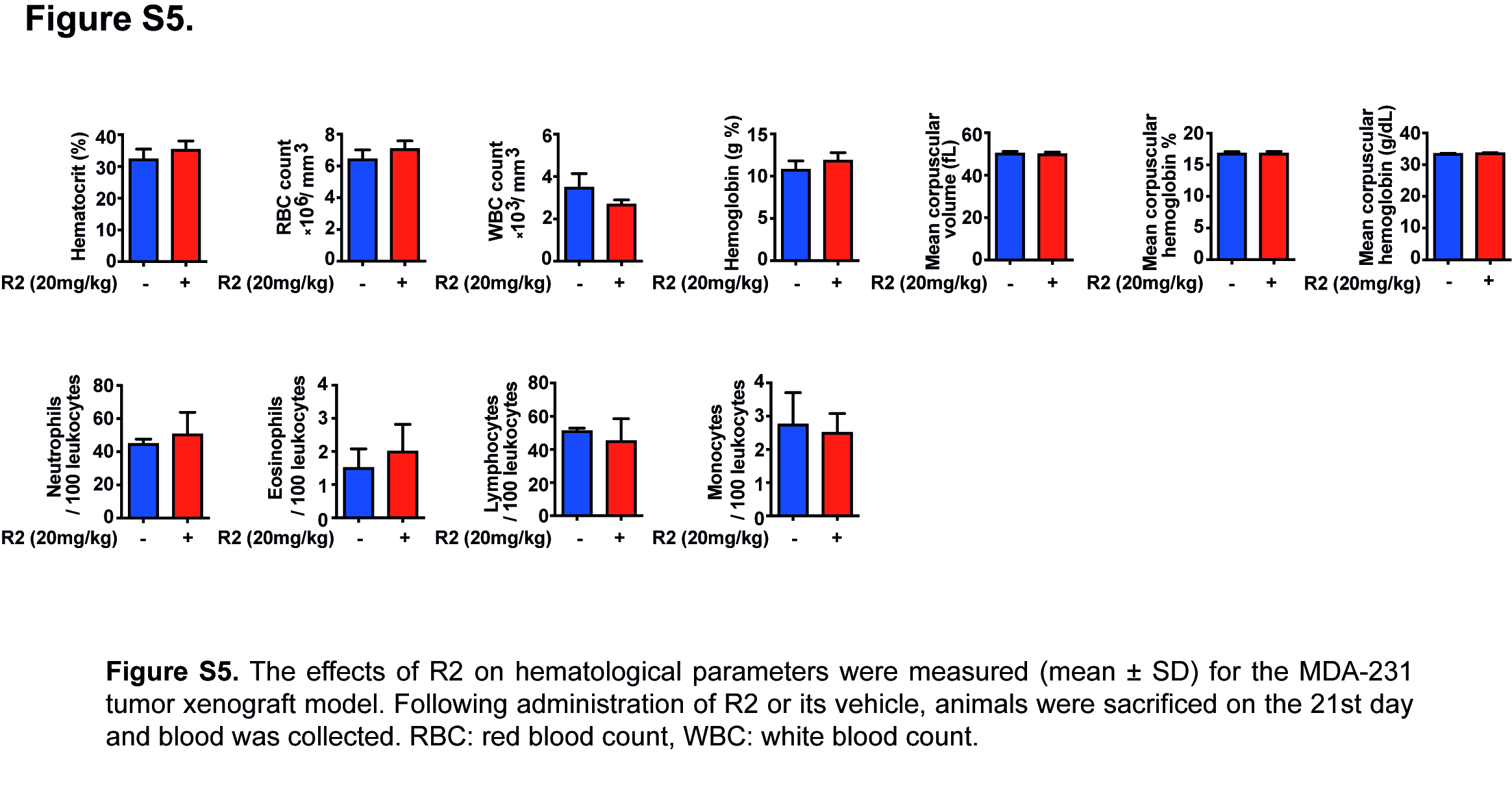

Supplement: Supplementary file 8 — Figure S5 [file 41419_2022_4855_MOESM8_ESM.tif]

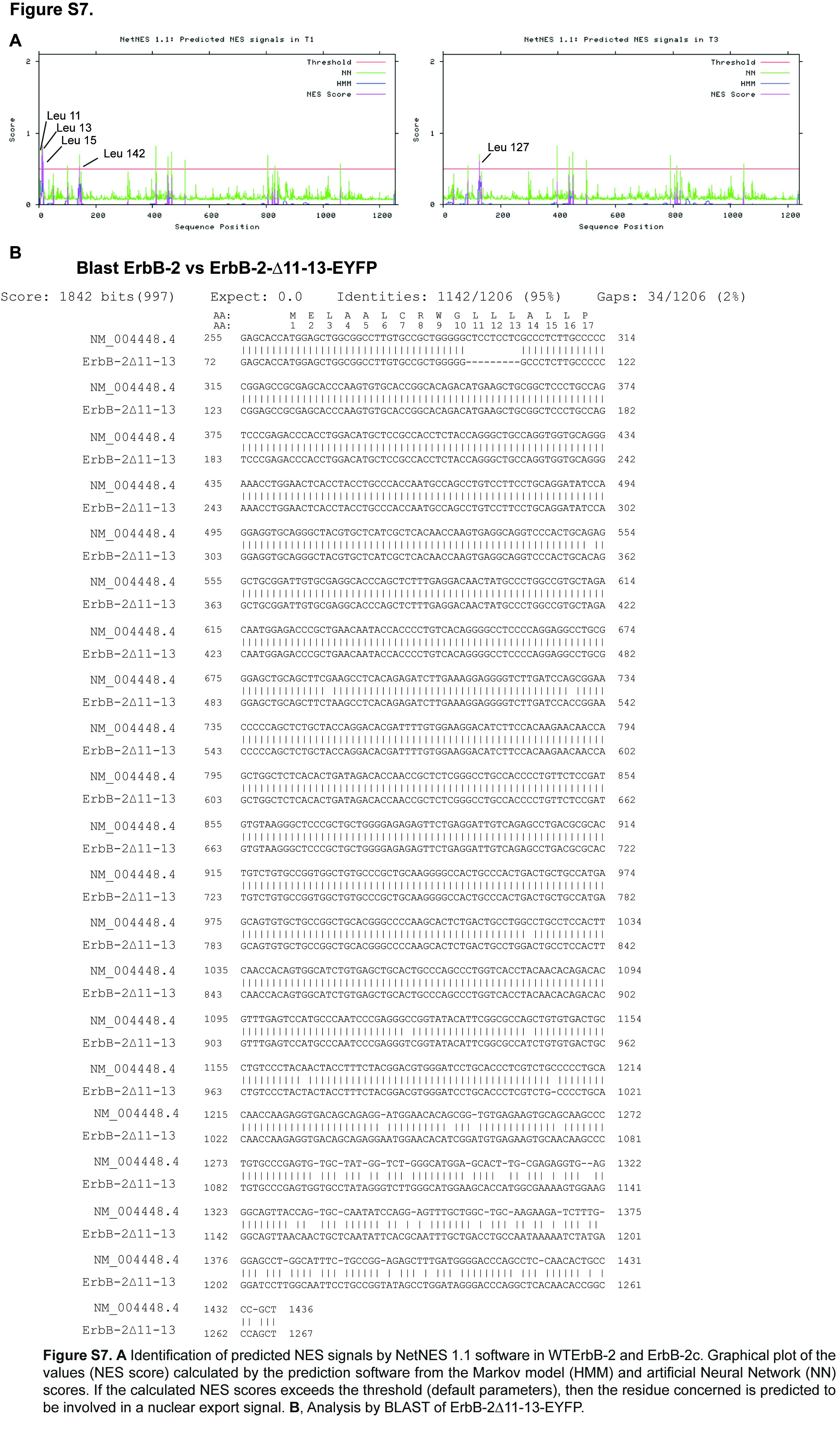

Supplement: Supplementary file 10 — Figure S7 [file 41419_2022_4855_MOESM10_ESM.tif]

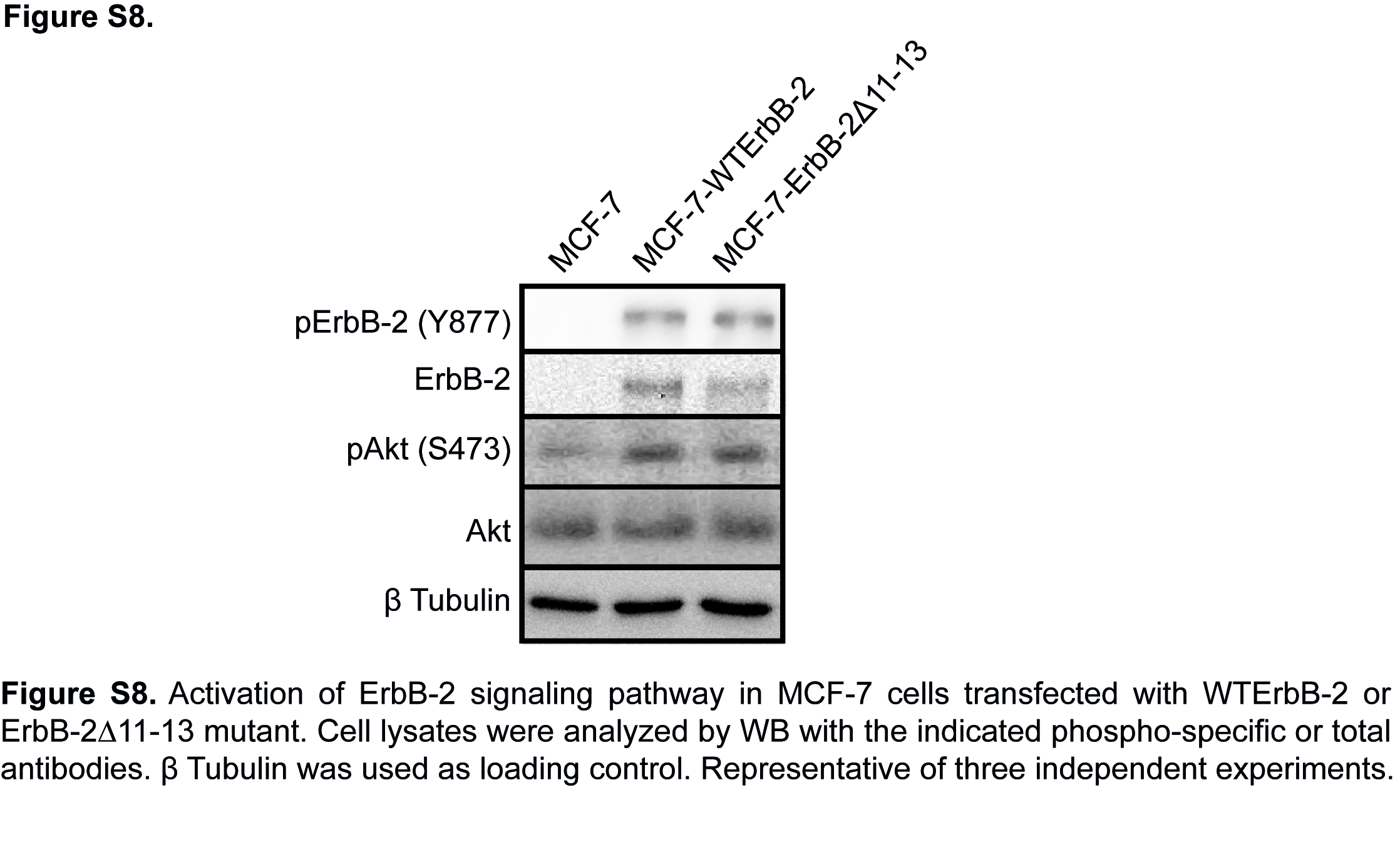

Supplement: Supplementary file 11 — Figure S8 [file 41419_2022_4855_MOESM11_ESM.tif]

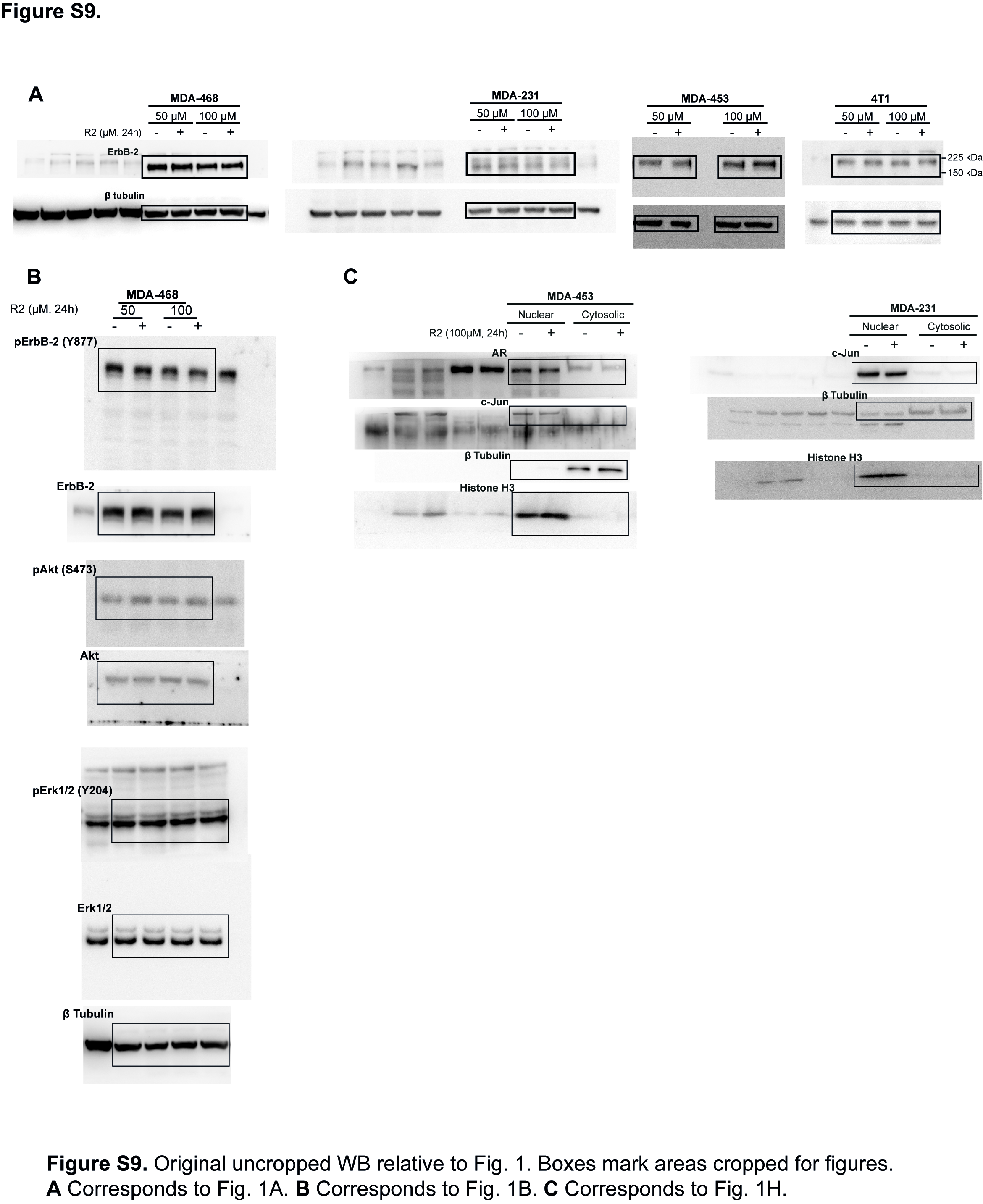

Supplement: Supplementary file 12 — Figure S9 [file 41419_2022_4855_MOESM12_ESM.tif]
