# Supplementary material for: Halting ErbB-2 isoforms retrograde transport to the nucleus as a new theragnostic approach for triple-negative breast cancer
Source: Cell Death Dis. 2022 May 9;13(5):447. doi: 10.1038/s41419-022-04855-0 (PMC9084267; doi:10.1038/s41419-022-04855-0)
Supplement: Supplementary file 9 — Figure S6 [file 41419_2022_4855_MOESM9_ESM.pdf]

Figure S6.

A BlastN 5'UTR sequence alignment

```
NM_004448.4   ErbB-2 Transcript 1 (T1)  Length: 175
1      cccctccatt gggaccggag aaaccagggg agccccccgg gcagccgcgc gccccttccc
61     acggggccct ttactgcgcc gcgcgcccgg cccccacccc tcgcagcacc ccgcgccccg
121    cgccctccca gccgggtcca gccggagcca tggggccgga gccgcagtga gcacc
```

```
NM_001289936.2 ErbB-2 Transcript 3 (T3)  Length: 572
1      gttctttatt ctactctccg ctgaagtcca cacagtttaa attaaagttc ccggattttt
61     gtgggcgcct gccccgcccc tcgtcccccct gctgtgtcca tatatcgagg cgatagggtt
121    aagggaaggc ggacgcctga tgggttaatg agcaaactga agtgttttcc atgatctttt
181    ttgagtcgca attgaagtac cacctcccga gggtgattgc ttccccatgc ggggtagaac
241    ctttgctgtc ctgttcacca ctctacctcc agcacagaat ttggcttatg cctactcaat
301    gtgaagatga tgaggatgaa aacctttgtg atgatccact tccacttaat gaatggtggc
361    aaagcaaagc tatattcaag accacatgca aagctactcc ctgagcaaag agtcacagat
421    aaaacggggg caccagtaga atggccagga caaacgcagt gcagcacaga gactcagacc
481    ctggcagcca tgcctgcgca ggcagtgatg agagtgacat gtactgttgt ggacatgcac
541    aaaagtgaga tacttcaaag attccagaag at
```

No significant similarity found.

B BlastN 3'UTR sequence alignment

Score: 1134 bits(614) Expect: 0.0 Query cover: 100% Identities: 614/614 (100%) Gaps: 0/614 (0%)

```
NM_004448.4     T1 1   ACCAGAAGGCCAAGTCCGCAGAAGCCCTGATGTGTCCTCAGGGAGCAGGGAAGGCCTGAC  60
                       ||||||||||||||||||||||||||||||||||||||||||||||||||||||||||||
NM_001289936.2  T3 1   ACCAGAAGGCCAAGTCCGCAGAAGCCCTGATGTGTCCTCAGGGAGCAGGGAAGGCCTGAC  60
NM_004448.4     T1 61  TTCTGCTGGCATCAAGAGGTGGGAGGGCCCTCCGACCACTTCCAGGGGAACCTGCCATGC  120
                       ||||||||||||||||||||||||||||||||||||||||||||||||||||||||||||
NM_001289936.2  T3 61  TTCTGCTGGCATCAAGAGGTGGGAGGGCCCTCCGACCACTTCCAGGGGAACCTGCCATGC  120
NM_004448.4     T1 121 CAGGAACCTGTCCTAAGGAACCTTCCTTCCTGCTTGAGTTCCCAGATGGCTGGAAGGGGT  180
                       ||||||||||||||||||||||||||||||||||||||||||||||||||||||||||||
NM_001289936.2  T3 121 CAGGAACCTGTCCTAAGGAACCTTCCTTCCTGCTTGAGTTCCCAGATGGCTGGAAGGGGT  180
NM_004448.4     T1 181 CCAGCCTCGTTGGAAGAGGAACAGCACTGGGGAGTCTTTGTGGATTCTGAGGCCCTGCCC  240
                       ||||||||||||||||||||||||||||||||||||||||||||||||||||||||||||
NM_001289936.2  T3 181 CCAGCCTCGTTGGAAGAGGAACAGCACTGGGGAGTCTTTGTGGATTCTGAGGCCCTGCCC  240
NM_004448.4     T1 241 AATGAGACTCTAGGGTCCAGTGGATGCCACAGCCCAGCTTGGCCCTTTCCTTCCAGATCC  300
                       ||||||||||||||||||||||||||||||||||||||||||||||||||||||||||||
NM_001289936.2  T3 241 AATGAGACTCTAGGGTCCAGTGGATGCCACAGCCCAGCTTGGCCCTTTCCTTCCAGATCC  300
NM_004448.4     T1 301 TGGGTACTGAAAGCCTTAGGGAAGCTGGCCTGAGAGGGGAAGCGGCCCTAAGGGAGTGTC  360
                       ||||||||||||||||||||||||||||||||||||||||||||||||||||||||||||
NM_001289936.2  T3 301 TGGGTACTGAAAGCCTTAGGGAAGCTGGCCTGAGAGGGGAAGCGGCCCTAAGGGAGTGTC  360
NM_004448.4     T1 361 TAAGAACAAAAGCGACCCATTCAGAGACTGTCCCTGAAACCTAGTACTGCCCCCCATGAG  420
                       ||||||||||||||||||||||||||||||||||||||||||||||||||||||||||||
NM_001289936.2  T3 361 TAAGAACAAAAGCGACCCATTCAGAGACTGTCCCTGAAACCTAGTACTGCCCCCCATGAG  420
NM_004448.4     T1 421 GAAGGAACAGCAATGGTGTCAGTATCCAGGCTTTGTACAGAGTGCttttctgtttagttt  480
                       ||||||||||||||||||||||||||||||||||||||||||||||||||||||||||||
NM_001289936.2  T3 421 GAAGGAACAGCAATGGTGTCAGTATCCAGGCTTTGTACAGAGTGCTTTTCTGTTTAGTTT  480
NM_004448.4     T1 481 ttactttttttgttttgtttttttAAAGATGAAATAAAGACCCAGGGGGAGAATGGGTGT  540
                       ||||||||||||||||||||||||||||||||||||||||||||||||||||||||||||
NM_001289936.2  T3 481 TTACTTTTTTTGTTTTGTTTTTTTAAAGATGAAATAAAGACCCAGGGGGAGAATGGGTGT  540
NM_004448.4     T1 541 TGTATGGGGAGGCAAGTGTGGGGGGTCCTTCTCCACACCCACTTTGTCCATTTGCAAATA  600
                       ||||||||||||||||||||||||||||||||||||||||||||||||||||||||||||
NM_001289936.2  T3 541 TGTATGGGGAGGCAAGTGTGGGGGGTCCTTCTCCACACCCACTTTGTCCATTTGCAAATA  600
NM_004448.4     T1 601 TATTTTGGAAAACA  614
                       ||||||||||||||
NM_001289936.2  T3 601 TATTTTGGAAAACA  614
```

C BlastN CCDSs sequence alignment

```
Score: 6831 bits(3699) Expect: 0.0 Query cover: 98% Identities: 3699/3699(100%) Gaps: 0/3699 (0%)

NM_004448.4      T1  1                                                    ATGGAGCTG  9
                                                                          .........
NM_004448.4      T1  10    GCGGCCTTGTGCCGCTGGGGGCTCCTCCTCGCCCTCTTGCCCCCCGGAGCCGCGAGCACC  69
NM_001289936.2   T3  1     ····································ATGCCCCGGGGGTCCTGGAAGCCA  24
NM_004448.4      T1  70    CAAGTGTGCACCGGCACAGACATGAAGCTGCGGCTCCCTGCCAGTCCCGAGACCCACCTG  129
                           ||||||||||||||||||||||||||||||||||||||||||||||||||||||||||||
NM_001289936.2   T3  25    CAAGTGTGCACCGGCACAGACATGAAGCTGCGGCTCCCTGCCAGTCCCGAGACCCACCTG  84
NM_004448.4      T1  130   GACATGCTCCGCCACCTCTACCAGGGCTGCCAGGTGGTGCAGGGAAACCTGGAACTCACC  189
                           ||||||||||||||||||||||||||||||||||||||||||||||||||||||||||||
NM_001289936.2   T3  85    GACATGCTCCGCCACCTCTACCAGGGCTGCCAGGTGGTGCAGGGAAACCTGGAACTCACC  144
NM_004448.4      T1  190   TACCTGCCCACCAATGCCAGCCTGTCCTTCCTGCAGGATATCCAGGAGGTGCAGGGCTAC  249
                           ||||||||||||||||||||||||||||||||||||||||||||||||||||||||||||
NM_001289936.2   T3  145   TACCTGCCCACCAATGCCAGCCTGTCCTTCCTGCAGGATATCCAGGAGGTGCAGGGCTAC  204
NM_004448.4      T1  250   GTGCTCATCGCTCACAACCAAGTGAGGCAGGTCCCACTGCAGAGGCTGCGGATTGTGCGA  309
                           ||||||||||||||||||||||||||||||||||||||||||||||||||||||||||||
NM_001289936.2   T3  205   GTGCTCATCGCTCACAACCAAGTGAGGCAGGTCCCACTGCAGAGGCTGCGGATTGTGCGA  264
NM_004448.4      T1   310  GGCACCCAGCTCTTTGAGGACAACTATGCCCTGGCCGTGCTAGACAATGGAGACCCGCTG  369
                           ||||||||||||||||||||||||||||||||||||||||||||||||||||||||||||
NM_001289936.2   T3   265  GGCACCCAGCTCTTTGAGGACAACTATGCCCTGGCCGTGCTAGACAATGGAGACCCGCTG  324
NM_004448.4      T1   370  AACAATACCACCCCTGTCACAGGGGCCTCCCCAGGAGGCCTGCGGGAGCTGCAGCTTCGA  429
                           ||||||||||||||||||||||||||||||||||||||||||||||||||||||||||||
NM_001289936.2   T3   325  AACAATACCACCCCTGTCACAGGGGCCTCCCCAGGAGGCCTGCGGGAGCTGCAGCTTCGA  384
NM_004448.4      T1   430  AGCCTCACAGAGATCTTGAAAGGAGGGGTCTTGATCCAGCGGAACCCCCAGCTCTGCTAC  489
                           ||||||||||||||||||||||||||||||||||||||||||||||||||||||||||||
NM_001289936.2   T3   385  AGCCTCACAGAGATCTTGAAAGGAGGGGTCTTGATCCAGCGGAACCCCCAGCTCTGCTAC  444
NM_004448.4      T1   490  CAGGACACGATTTTGTGGAAGGACATCTTCCACAAGAACAACCAGCTGGCTCTCACACTG  549
                           ||||||||||||||||||||||||||||||||||||||||||||||||||||||||||||
NM_001289936.2   T3   445  CAGGACACGATTTTGTGGAAGGACATCTTCCACAAGAACAACCAGCTGGCTCTCACACTG  504
NM_004448.4      T1   550  ATAGACACCAACCGCTCTCGGGCCTGCCACCCCTGTTCTCCGATGTGTAAGGGCTCCCGC  609
                           ||||||||||||||||||||||||||||||||||||||||||||||||||||||||||||
NM_001289936.2   T3   505  ATAGACACCAACCGCTCTCGGGCCTGCCACCCCTGTTCTCCGATGTGTAAGGGCTCCCGC  564
NM_004448.4      T1   610  TGCTGGGGAGAGAGTTCTGAGGATTGTCAGAGCCTGACGCGCACTGTCTGTGCCGGTGGC  669
                           ||||||||||||||||||||||||||||||||||||||||||||||||||||||||||||
NM_001289936.2   T3   565  TGCTGGGGAGAGAGTTCTGAGGATTGTCAGAGCCTGACGCGCACTGTCTGTGCCGGTGGC  624
NM_004448.4      T1   670  TGTGCCCGCTGCAAGGGGCCACTGCCCACTGACTGCTGCCATGAGCAGTGTGCTGCCGGC  729
                           ||||||||||||||||||||||||||||||||||||||||||||||||||||||||||||
NM_001289936.2   T3   625  TGTGCCCGCTGCAAGGGGCCACTGCCCACTGACTGCTGCCATGAGCAGTGTGCTGCCGGC  684
NM_004448.4      T1   730  TGCACGGGCCCCAAGCACTCTGACTGCCTGGCCTGCCTCCACTTCAACCACAGTGGCATC  789
                           ||||||||||||||||||||||||||||||||||||||||||||||||||||||||||||
NM_001289936.2   T3   685  TGCACGGGCCCCAAGCACTCTGACTGCCTGGCCTGCCTCCACTTCAACCACAGTGGCATC  744
NM_004448.4      T1   790  TGTGAGCTGCACTGCCCAGCCCTGGTCACCTACAACACAGACACGTTTGAGTCCATGCCC  849
                           ||||||||||||||||||||||||||||||||||||||||||||||||||||||||||||
NM_001289936.2   T3   745  TGTGAGCTGCACTGCCCAGCCCTGGTCACCTACAACACAGACACGTTTGAGTCCATGCCC  804
NM_004448.4      T1   850  AATCCCGAGGGCCGGTATACATTCGGCGCCAGCTGTGTGACTGCCTGTCCCTACAACTAC  909
                           ||||||||||||||||||||||||||||||||||||||||||||||||||||||||||||
NM_001289936.2   T3   805  AATCCCGAGGGCCGGTATACATTCGGCGCCAGCTGTGTGACTGCCTGTCCCTACAACTAC  864
NM_004448.4      T1   910  CTTTCTACGGACGTGGGATCCTGCACCCTCGTCTGCCCCCTGCACAACCAAGAGGTGACA  969
                           ||||||||||||||||||||||||||||||||||||||||||||||||||||||||||||
NM_001289936.2   T3   865  CTTTCTACGGACGTGGGATCCTGCACCCTCGTCTGCCCCCTGCACAACCAAGAGGTGACA  924
NM_004448.4      T1   970  GCAGAGGATGGAACACAGCGGTGTGAGAAGTGCAGCAAGCCCTGTGCCCGAGTGTGCTAT  1029
                           ||||||||||||||||||||||||||||||||||||||||||||||||||||||||||||
NM_001289936.2   T3   925  GCAGAGGATGGAACACAGCGGTGTGAGAAGTGCAGCAAGCCCTGTGCCCGAGTGTGCTAT  984
NM_004448.4      T1   1030 GGTCTGGGCATGGAGCACTTGCGAGAGGTGAGGGCAGTTACCAGTGCCAATATCCAGGAG  1089
                           ||||||||||||||||||||||||||||||||||||||||||||||||||||||||||||
NM_001289936.2   T3   985  GGTCTGGGCATGGAGCACTTGCGAGAGGTGAGGGCAGTTACCAGTGCCAATATCCAGGAG  1044
NM_004448.4      T1   1090 TTTGCTGGCTGCAAGAAGATCTTTGGGAGCCTGGCATTTCTGCCGGAGAGCTTTGATGGG  1149
                           ||||||||||||||||||||||||||||||||||||||||||||||||||||||||||||
NM_001289936.2   T3   1045 TTTGCTGGCTGCAAGAAGATCTTTGGGAGCCTGGCATTTCTGCCGGAGAGCTTTGATGGG  1104
NM_004448.4      T1   1150 GACCCAGCCTCCAACACTGCCCCGCTCCAGCCAGAGCAGCTCCAAGTGTTTGAGACTCTG  1209
                           ||||||||||||||||||||||||||||||||||||||||||||||||||||||||||||
NM_001289936.2   T3   1105 GACCCAGCCTCCAACACTGCCCCGCTCCAGCCAGAGCAGCTCCAAGTGTTTGAGACTCTG  1164
```

```
NM_004448.4      T1    1210  GAAGAGATCACAGGTTACCTATACATCTCAGCATGGCCGGACAGCCTGCCTGACCTCAGC  1269
                             ||||||||||||||||||||||||||||||||||||||||||||||||||||||||||||
NM_001289936.2   T3    1165  GAAGAGATCACAGGTTACCTATACATCTCAGCATGGCCGGACAGCCTGCCTGACCTCAGC  1224
NM_004448.4      T1    1270  GTCTTCCAGAACCTGCAAGTAATCCGGGGACGAATTCTGCACAATGGCGCCTACTCGCTG  1329
                             ||||||||||||||||||||||||||||||||||||||||||||||||||||||||||||
NM_001289936.2   T3    1225  GTCTTCCAGAACCTGCAAGTAATCCGGGGACGAATTCTGCACAATGGCGCCTACTCGCTG  1284
NM_004448.4      T1    1330  ACCCTGCAAGGGCTGGGCATCAGCTGGCTGGGGCTGCGCTCACTGAGGGAACTGGGCAGT  1389
                             ||||||||||||||||||||||||||||||||||||||||||||||||||||||||||||
NM_001289936.2   T3    1285  ACCCTGCAAGGGCTGGGCATCAGCTGGCTGGGGCTGCGCTCACTGAGGGAACTGGGCAGT  1344
NM_004448.4      T1    1390  GGACTGGCCCTCATCCACCATAACACCCACCTCTGCTTCGTGCACACGGTGCCCTGGGAC  1449
                             ||||||||||||||||||||||||||||||||||||||||||||||||||||||||||||
NM_001289936.2   T3    1345  GGACTGGCCCTCATCCACCATAACACCCACCTCTGCTTCGTGCACACGGTGCCCTGGGAC  1404
NM_004448.4      T1    1450  CAGCTCTTTCGGAACCCGCACCAAGCTCTGCTCCACACTGCCAACCGGCCAGAGGACGAG  1509
                             ||||||||||||||||||||||||||||||||||||||||||||||||||||||||||||
NM_001289936.2   T3    1405  CAGCTCTTTCGGAACCCGCACCAAGCTCTGCTCCACACTGCCAACCGGCCAGAGGACGAG  1464
NM_004448.4      T1    1510  TGTGTGGGCGAGGGCCTGGCCTGCCACCAGCTGTGCGCCCGAGGGCACTGCTGGGGTCCA  1569
                             ||||||||||||||||||||||||||||||||||||||||||||||||||||||||||||
NM_001289936.2   T3    1465  TGTGTGGGCGAGGGCCTGGCCTGCCACCAGCTGTGCGCCCGAGGGCACTGCTGGGGTCCA  1524
NM_004448.4      T1    1570  GGGCCCACCCAGTGTGTCAACTGCAGCCAGTTCCTTCGGGGCCAGGAGTGCGTGGAGGAA  1629
                             ||||||||||||||||||||||||||||||||||||||||||||||||||||||||||||
NM_001289936.2   T3    1525  GGGCCCACCCAGTGTGTCAACTGCAGCCAGTTCCTTCGGGGCCAGGAGTGCGTGGAGGAA  1584
NM_004448.4      T1    1630  TGCCGAGTACTGCAGGGGCTCCCCAGGGAGTATGTGAATGCCAGGCACTGTTTGCCGTGC  1689
                             ||||||||||||||||||||||||||||||||||||||||||||||||||||||||||||
NM_001289936.2   T3    1585  TGCCGAGTACTGCAGGGGCTCCCCAGGGAGTATGTGAATGCCAGGCACTGTTTGCCGTGC  1644
NM_004448.4      T1    1690  CACCCTGAGTGTCAGCCCCAGAATGGCTCAGTGACCTGTTTTGGACCGGAGGCTGACCAG  1749
                             ||||||||||||||||||||||||||||||||||||||||||||||||||||||||||||
NM_001289936.2   T3    1645  CACCCTGAGTGTCAGCCCCAGAATGGCTCAGTGACCTGTTTTGGACCGGAGGCTGACCAG  1704
NM_004448.4      T1    1750  TGTGTGGCCTGTGCCCACTATAAGGACCCTCCCTTCTGCGTGGCCCGCTGCCCCAGCGGT  1809
                             ||||||||||||||||||||||||||||||||||||||||||||||||||||||||||||
NM_001289936.2   T3    1705  TGTGTGGCCTGTGCCCACTATAAGGACCCTCCCTTCTGCGTGGCCCGCTGCCCCAGCGGT  1764
NM_004448.4      T1    1810  GTGAAACCTGACCTCTCCTACATGCCCATCTGGAAGTTTCCAGATGAGGAGGGCGCATGC  1869
                             ||||||||||||||||||||||||||||||||||||||||||||||||||||||||||||
NM_001289936.2   T3    1765  GTGAAACCTGACCTCTCCTACATGCCCATCTGGAAGTTTCCAGATGAGGAGGGCGCATGC  1824
NM_004448.4      T1    1870  CAGCCTTGCCCCATCAACTGCACCCACTCCTGTGTGGACCTGGATGACAAGGGCTGCCCC  1929
                             ||||||||||||||||||||||||||||||||||||||||||||||||||||||||||||
NM_001289936.2   T3    1825  CAGCCTTGCCCCATCAACTGCACCCACTCCTGTGTGGACCTGGATGACAAGGGCTGCCCC  1884
NM_004448.4      T1    1930  GCCGAGCAGAGAGCCAGCCCTCTGACGTCCATCATCTCTGCGGTGGTTGGCATTCTGCTG  1989
                             ||||||||||||||||||||||||||||||||||||||||||||||||||||||||||||
NM_001289936.2   T3    1885  GCCGAGCAGAGAGCCAGCCCTCTGACGTCCATCATCTCTGCGGTGGTTGGCATTCTGCTG  1944
NM_004448.4      T1    1990  GTCGTGGTCTTGGGGGTGGTCTTTGGGATCCTCATCAAGCGACGGCAGCAGAAGATCCGG  2049
                             ||||||||||||||||||||||||||||||||||||||||||||||||||||||||||||
NM_001289936.2   T3    1945  GTCGTGGTCTTGGGGGTGGTCTTTGGGATCCTCATCAAGCGACGGCAGCAGAAGATCCGG  2004
NM_004448.4      T1    2050  AAGTACACGATGCGGAGACTGCTGCAGGAAACGGAGCTGGTGGAGCCGCTGACACCTAGC  2109
                             ||||||||||||||||||||||||||||||||||||||||||||||||||||||||||||
NM_001289936.2   T3    2005  AAGTACACGATGCGGAGACTGCTGCAGGAAACGGAGCTGGTGGAGCCGCTGACACCTAGC  2064
NM_004448.4      T1    2110  GGAGCGATGCCCAACCAGGCGCAGATGCGGATCCTGAAAGAGACGGAGCTGAGGAAGGTG  2169
                             ||||||||||||||||||||||||||||||||||||||||||||||||||||||||||||
NM_001289936.2   T3    2065  GGAGCGATGCCCAACCAGGCGCAGATGCGGATCCTGAAAGAGACGGAGCTGAGGAAGGTG  2124
NM_004448.4      T1    2170  AAGGTGCTTGGATCTGGCGCTTTTGGCACAGTCTACAAGGGCATCTGGATCCCTGATGGG  2229
                             ||||||||||||||||||||||||||||||||||||||||||||||||||||||||||||
NM_001289936.2   T3    2125  AAGGTGCTTGGATCTGGCGCTTTTGGCACAGTCTACAAGGGCATCTGGATCCCTGATGGG  2184
NM_004448.4      2230        GAGAATGTGAAAATTCCAGTGGCCATCAAAGTGTTGAGGGAAAACACATCCCCCAAAGCC  2289
                             ||||||||||||||||||||||||||||||||||||||||||||||||||||||||||||
NM_001289936.2   T3    2185  GAGAATGTGAAAATTCCAGTGGCCATCAAAGTGTTGAGGGAAAACACATCCCCCAAAGCC  2244
NM_004448.4      T1    2290  AACAAAGAAATCTTAGACGAAGCATACGTGATGGCTGGTGTGGGCTCCCCATATGTCTCC  2349
                             ||||||||||||||||||||||||||||||||||||||||||||||||||||||||||||
NM_001289936.2   T3    2245  AACAAAGAAATCTTAGACGAAGCATACGTGATGGCTGGTGTGGGCTCCCCATATGTCTCC  2304
NM_004448.4      T1    2350  CGCCTTCTGGGCATCTGCCTGACATCCACGGTGCAGCTGGTGACACAGCTTATGCCCTAT  2409
                             ||||||||||||||||||||||||||||||||||||||||||||||||||||||||||||
NM_001289936.2   T3    2305  CGCCTTCTGGGCATCTGCCTGACATCCACGGTGCAGCTGGTGACACAGCTTATGCCCTAT  2364
NM_004448.4      T1    2410  GGCTGCCTCTTAGACCATGTCCGGGAAAACCGCGGACGCCTGGGCTCCCAGGACCTGCTG  2469
                             ||||||||||||||||||||||||||||||||||||||||||||||||||||||||||||
NM_001289936.2   T3    2365  GGCTGCCTCTTAGACCATGTCCGGGAAAACCGCGGACGCCTGGGCTCCCAGGACCTGCTG  2424
NM_004448.4      T1    2470  AACTGGTGTATGCAGATTGCCAAGGGGATGAGCTACCTGGAGGATGTGCGGCTCGTACAC  2529
                             ||||||||||||||||||||||||||||||||||||||||||||||||||||||||||||
NM_001289936.2   T3    2425  AACTGGTGTATGCAGATTGCCAAGGGGATGAGCTACCTGGAGGATGTGCGGCTCGTACAC  2484
```

```
NM_004448.4      T1   2530  AGGGACTTGGCCGCTCGGAACGTGCTGGTCAAGAGTCCCAACCATGTCAAAATTACAGAC  2589
                            ||||||||||||||||||||||||||||||||||||||||||||||||||||||||||||
NM_001289936.2   T3   2485  AGGGACTTGGCCGCTCGGAACGTGCTGGTCAAGAGTCCCAACCATGTCAAAATTACAGAC  2544
NM_004448.4      T1   2590  TTCGGGCTGGCTCGGCTGCTGGACATTGACGAGACAGAGTACCATGCAGATGGGGGCAAG  2649
                            ||||||||||||||||||||||||||||||||||||||||||||||||||||||||||||
NM_001289936.2   T3   2545  TTCGGGCTGGCTCGGCTGCTGGACATTGACGAGACAGAGTACCATGCAGATGGGGGCAAG  2604
NM_004448.4      T1   2650  GTGCCCATCAAGTGGATGGCGCTGGAGTCCATTCTCCGCCGGCGGTTCACCCACCAGAGT  2709
                            ||||||||||||||||||||||||||||||||||||||||||||||||||||||||||||
NM_001289936.2   T3   2605  GTGCCCATCAAGTGGATGGCGCTGGAGTCCATTCTCCGCCGGCGGTTCACCCACCAGAGT  2664
NM_004448.4      T1   2710  GATGTGTGGAGTTATGGTGTGACTGTGTGGGAGCTGATGACTTTTGGGGCCAAACCTTAC  2769
                            ||||||||||||||||||||||||||||||||||||||||||||||||||||||||||||
NM_001289936.2   T3   2665  GATGTGTGGAGTTATGGTGTGACTGTGTGGGAGCTGATGACTTTTGGGGCCAAACCTTAC  2724
NM_004448.4      T1   2770  GATGGGATCCCAGCCCGGGAGATCCCTGACCTGCTGGAAAAGGGGGAGCGGCTGCCCCAG  2829
                            ||||||||||||||||||||||||||||||||||||||||||||||||||||||||||||
NM_001289936.2   T3   2725  GATGGGATCCCAGCCCGGGAGATCCCTGACCTGCTGGAAAAGGGGGAGCGGCTGCCCCAG  2784
NM_004448.4      T1   2830  CCCCCCATCTGCACCATTGATGTCTACATGATCATGGTCAAATGTTGGATGATTGACTCT  2889
                            ||||||||||||||||||||||||||||||||||||||||||||||||||||||||||||
NM_001289936.2   T3   2785  CCCCCCATCTGCACCATTGATGTCTACATGATCATGGTCAAATGTTGGATGATTGACTCT  2844
NM_004448.4      T1   2890  GAATGTCGGCCAAGATTCCGGGAGTTGGTGTCTGAATTCTCCCGCATGGCCAGGGACCCC  2949
                            ||||||||||||||||||||||||||||||||||||||||||||||||||||||||||||
NM_001289936.2   T3   2845  GAATGTCGGCCAAGATTCCGGGAGTTGGTGTCTGAATTCTCCCGCATGGCCAGGGACCCC  2904
NM_004448.4      T1   2950  CAGCGCTTTGTGGTCATCCAGAATGAGGACTTGGGCCCAGCCAGTCCCTTGGACAGCACC  3009
                            ||||||||||||||||||||||||||||||||||||||||||||||||||||||||||||
NM_001289936.2   T3   2905  CAGCGCTTTGTGGTCATCCAGAATGAGGACTTGGGCCCAGCCAGTCCCTTGGACAGCACC  2964
NM_004448.4      T1   3010  TTCTACCGCTCACTGCTGGAGGACGATGACATGGGGGACCTGGTGGATGCTGAGGAGTAT  3069
                            ||||||||||||||||||||||||||||||||||||||||||||||||||||||||||||
NM_001289936.2   T3   2965  TTCTACCGCTCACTGCTGGAGGACGATGACATGGGGGACCTGGTGGATGCTGAGGAGTAT  3024
NM_004448.4      T1   3070  CTGGTACCCCAGCAGGGCTTCTTCTGTCCAGACCCTGCCCCGGGCGCTGGGGGCATGGTC  3129
                            ||||||||||||||||||||||||||||||||||||||||||||||||||||||||||||
NM_001289936.2   T3   3025  CTGGTACCCCAGCAGGGCTTCTTCTGTCCAGACCCTGCCCCGGGCGCTGGGGGCATGGTC  3084
NM_004448.4      T1   3130  CACCACAGGCACCGCAGCTCATCTACCAGGAGTGGCGGTGGGGACCTGACACTAGGGCTG  3189
                            ||||||||||||||||||||||||||||||||||||||||||||||||||||||||||||
NM_001289936.2   T3   3085  CACCACAGGCACCGCAGCTCATCTACCAGGAGTGGCGGTGGGGACCTGACACTAGGGCTG  3144
NM_004448.4      T1   3190  GAGCCCTCTGAAGAGGAGGCCCCCAGGTCTCCACTGGCACCCTCCGAAGGGGCTGGCTCC  3249
                            ||||||||||||||||||||||||||||||||||||||||||||||||||||||||||||
NM_001289936.2   T3   3145  GAGCCCTCTGAAGAGGAGGCCCCCAGGTCTCCACTGGCACCCTCCGAAGGGGCTGGCTCC  3204
NM_004448.4      T1   3250  GATGTATTTGATGGTGACCTGGGAATGGGGGCAGCCAAGGGGCTGCAAAGCCTCCCCACA  3309
                            ||||||||||||||||||||||||||||||||||||||||||||||||||||||||||||
NM_001289936.2   T3   3205  GATGTATTTGATGGTGACCTGGGAATGGGGGCAGCCAAGGGGCTGCAAAGCCTCCCCACA  3264
NM_004448.4      T1   3310  CATGACCCCAGCCCTCTACAGCGGTACAGTGAGGACCCCACAGTACCCCTGCCCTCTGAG  3369
                            ||||||||||||||||||||||||||||||||||||||||||||||||||||||||||||
NM_001289936.2   T3   3265  CATGACCCCAGCCCTCTACAGCGGTACAGTGAGGACCCCACAGTACCCCTGCCCTCTGAG  3324
NM_004448.4      T1   3370  ACTGATGGCTACGTTGCCCCCCTGACCTGCAGCCCCCAGCCTGAATATGTGAACCAGCCA  3429
                            ||||||||||||||||||||||||||||||||||||||||||||||||||||||||||||
NM_001289936.2   T3   3325  ACTGATGGCTACGTTGCCCCCCTGACCTGCAGCCCCCAGCCTGAATATGTGAACCAGCCA  3384
NM_004448.4      T1   3430  GATGTTCGGCCCCAGCCCCCTTCGCCCCGAGAGGGCCCTCTGCCTGCTGCCCGACCTGCT  3489
                            ||||||||||||||||||||||||||||||||||||||||||||||||||||||||||||
NM_001289936.2   T3   3385  GATGTTCGGCCCCAGCCCCCTTCGCCCCGAGAGGGCCCTCTGCCTGCTGCCCGACCTGCT  3444
NM_004448.4      T1   3490  GGTGCCACTCTGGAAAGGCCCAAGACTCTCTCCCCAGGGAAGAATGGGGTCGTCAAAGAC  3549
                            ||||||||||||||||||||||||||||||||||||||||||||||||||||||||||||
NM_001289936.2   T3   3445  GGTGCCACTCTGGAAAGGCCCAAGACTCTCTCCCCAGGGAAGAATGGGGTCGTCAAAGAC  3504
NM_004448.4      T1   3550  GTTTTTGCCTTTGGGGGTGCCGTGGAGAACCCCGAGTACTTGACACCCCAGGGAGGAGCT  3609
                            ||||||||||||||||||||||||||||||||||||||||||||||||||||||||||||
NM_001289936.2   T3   3505  GTTTTTGCCTTTGGGGGTGCCGTGGAGAACCCCGAGTACTTGACACCCCAGGGAGGAGCT  3564
NM_004448.4      T1   3610  GCCCCTCAGCCCCACCCTCCTCCTGCCTTCAGCCCAGCCTTCGACAACCTCTATTACTGG  3669
                            ||||||||||||||||||||||||||||||||||||||||||||||||||||||||||||
NM_001289936.2   T3   3565  GCCCCTCAGCCCCACCCTCCTCCTGCCTTCAGCCCAGCCTTCGACAACCTCTATTACTGG  3624
NM_004448.4      T1   3670  GACCAGGACCCACCAGAGCGGGGGGCTCCACCCAGCACCTTCAAAGGGACACCTACGGCA  3729
                            ||||||||||||||||||||||||||||||||||||||||||||||||||||||||||||
NM_001289936.2   T3   3625  GACCAGGACCCACCAGAGCGGGGGGCTCCACCCAGCACCTTCAAAGGGACACCTACGGCA  3684
NM_004448.4      T1   3730  GAGAACCCAGAGTACCTGGGTCTGGACGTGCCAGTGTGA  3768
                            |||||||||||||||||||||||||||||||||||||||
NM_001289936.2   T3   3685  GAGAACCCAGAGTACCTGGGTCTGGACGTGCCAGTGTGA  3723
```

Figure S6. Identity analysis of human T1 and T3. A and B, Analysis by BLAST showed that the 5'UTR of T1 (572 nt) had no significant similarity to that of T3 (175 nt) (A), whilst the 3'UTRs (614 nt) were conserved between the transcripts (100% nucleotide identity) (B). (See ref. [1]). Blue lines denote polyA signal sequences. (C) Analysis by BLAST showed 100% nucleotide identity in 98% of the T1 CCDS sequence covered by the T3 CCDS sequence.
