# Supplementary figures and images for: Correction: Halting ErbB-2 isoforms retrograde transport to the nucleus as a new theragnostic approach for triple-negative breast cancer
Source: Cell Death Dis. 2023 Dec 15;14(12):833. doi: 10.1038/s41419-023-06339-1 (PMC10724149; doi:10.1038/s41419-023-06339-1)

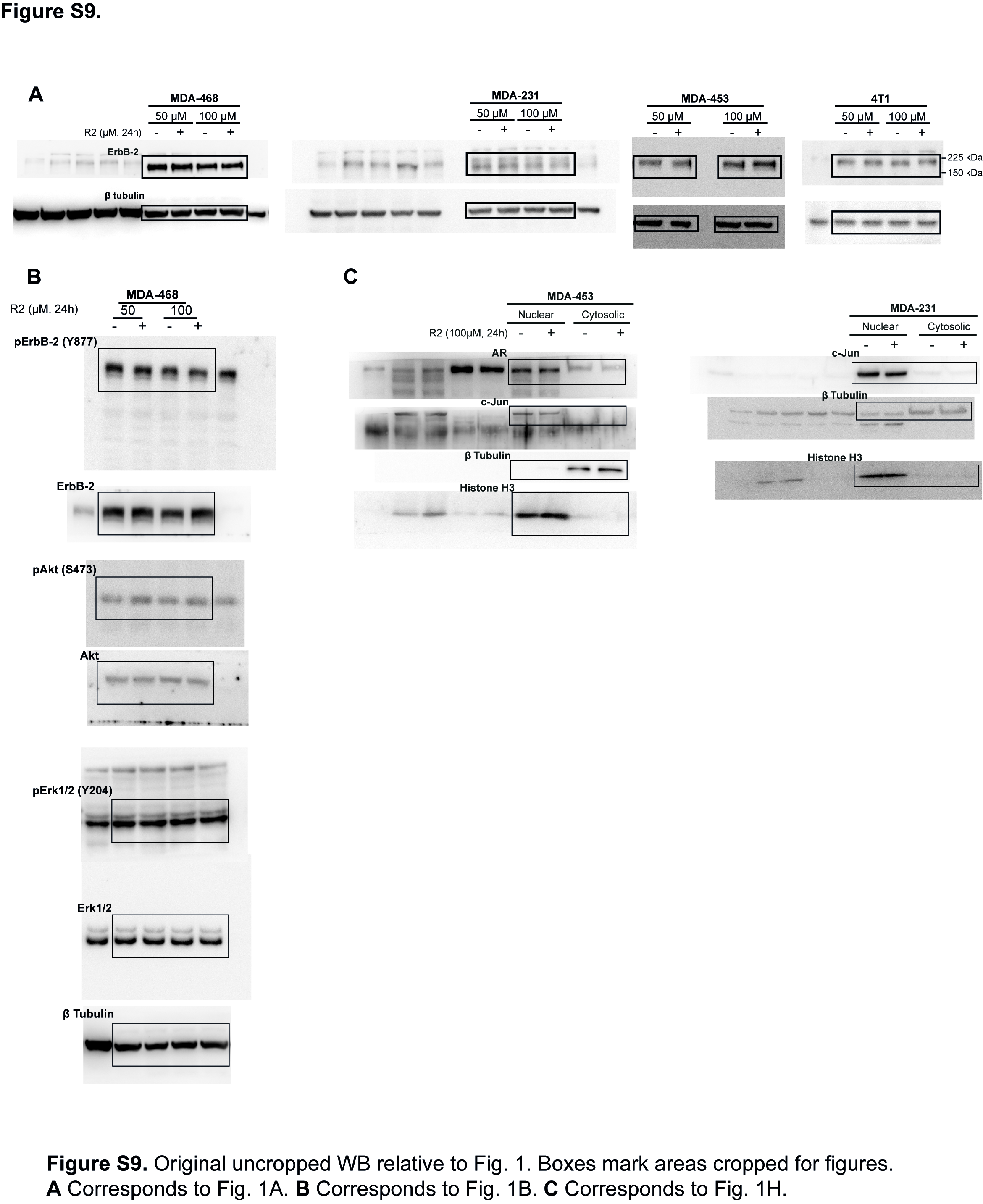

Supplement: Supplementary file 1 — Corrected_Supplementary_Figure 9 [file 41419_2023_6339_MOESM1_ESM.tif]
